# Palliating Salt Stress in Mustard through Plant-Growth-Promoting Rhizobacteria: Regulation of Secondary Metabolites, Osmolytes, Antioxidative Enzymes and Stress Ethylene

**DOI:** 10.3390/plants12040705

**Published:** 2023-02-05

**Authors:** Varisha Khan, Shahid Umar, Noushina Iqbal

**Affiliations:** Department of Botany, Jamia Hamdard, New Delhi 110062, India

**Keywords:** bioactive compounds, ethylene, oxidative stress, reduced glutathione

## Abstract

The severity of salt stress is alarming for crop growth and production and it threatens food security. Strategies employed for the reduction in stress are not always eco-friendly or sustainable. Plant-growth-promoting rhizobacteria (PGPR) could provide an alternative sustainable stress reduction strategy owning to its role in various metabolic processes. In this study, we have used two strains of PGPR, *Pseudomonas fluorescens* (NAIMCC-B-00340) and *Azotobacter chroococcum* Beijerinck 1901 (MCC 2351), either singly or in combination, and studied their effect in the amelioration of salt toxicity in mustard cultivar Pusa Jagannath via its influence on plants’ antioxidants’ metabolism, photosynthesis and growth. Individually, the impact of *Pseudomonas fluorescens* was better in reducing stress ethylene, oxidative stress, photosynthesis and growth but maximal alleviation was observed with their combined application. MDA and H_2_O_2_ content as indicator of oxidative stress decreased by 27.86% and 45.18% and osmolytes content (proline and glycine-betaine) increased by 38.8% and 26.3%, respectively, while antioxidative enzymes (SOD, CAT, APX and GR) increased by 58.40, 25.65, 81.081 and 55.914%, respectively, over salt-treated plants through the application of *Pseudomonas fluorescens*. The combined application maximally resulted in more cell viability and less damage to the leaf with lesser superoxide generation due to higher antioxidative enzymes and reduced glutathione formation (GSH). Considering the obtained results, we can supplement the PGPR in combination to plants subjected to salt stress, prevent photosynthetic and growth reduction, and increase the yield of plants.

## 1. Introduction

Any type of environmental factor that hampers plant growth and development can be considered an abiotic stress. Salinity stress is one of the prime abiotic stresses that causes huge productivity loss every year [1]. It affects around 954 million ha of land, which is approximately 25–30% worldwide land and about half of the total arable land irrigated globally [2].

The impact of salinity on plant growth is primarily through hyper-osmotic conditions; as stress progresses, the plant faces ion toxicity. This ionic and osmotic imbalance impairs plant growth and development [3,4], causing destructive effects on the morphological, physiological, and biochemical parameters of plants [5]. Wilting, necrosis, chlorosis, poor root and shoot growth, and eventually patchy and stunted plant growth are morphological changes that occur when plants are exposed to saline conditions [6]. Photosynthetic machinery inhibition, changes in transpiration and gaseous exchange via decreasing chlorophyll and carotenoids concentration, modifying chloroplast ultrastructure and PSII system, lowering stomatal conductance, increased Na^+^ transport and decreased K^+^ transport causing ion toxicity are some of the physiological and biochemical impacts on plants exposed to saline conditions [7]. High salinity causes excessive generation of reactive oxygen species (ROS), which are either partially reduced (such as superoxide radicals, hydrogen peroxide, and hydroxyl radicals) or excited (such as singlet oxygen) forms that cause oxidative modifications such as lipid peroxidation, protein oxidation and nucleic acid damage [8]. To resist cytosolic ROS toxicity, plants require an effective antioxidant defence mechanism (enzymatic and non-enzymatic antioxidants) that increases plant tolerance [9,10,11]. Strategic improvement of the antioxidant metabolism together with other stress busters, which include osmolytes and various secondary metabolites, could provide an effective method for salt tolerance. Major studies focus on the use of the plant growth regulators (PGRs) treatment, 24-epibrassinolide [12] or abscisic acid (ABA) analogs [13], improvement of mineral fertilization [14], and modification of gene expression [15,16] for plant salt tolerance. However, incorporating beneficial microbes, such as arbuscular mycorrhizal fungi or plant growth-promoting rhizobacteria (PGPR), to improve stress resistance is a trending concept [17] that is more eco-friendly and sustainable.

PGPR are bacteria that colonise the roots surrounding the rhizosphere, enhancing plant growth and development through a variety of different mechanisms. These PGPR stimulate the release of osmolytes such as proline, choline, trehalose, and increase nutrients’ availability through nitrogen fixation [18], phosphorous and potassium solubilisation [19,20] and iron sequestration [21]. Moreover, PGPR induce root growth and nutrient uptake by releasing phytohormones and secondary metabolites into the rhizosphere [22], and increase bacterial 1-aminocyclopropane-1-carboxylic acid (ACC)-deaminase activity in the rhizosphere by reducing ACC levels and ethylene in plant tissues [23,24,25].

Mustard (*Brassica juncea*) is an annual herb [26] occupying third place among the various oilseed species due to its considerable economic and nutritional value. Glucosinolates, flavonoids, anthocyanins, ß-carotene, and ascorbic acid are some of the bioactive compounds that contribute to its nutritional value [27]. Not only the leaves, but its seeds are also rich in protein, carbohydrates, dietary fibre, fats as well as vitamins (C and K), several trace minerals (Ca, Fe, Zn, Se, Cu, Mn, Mg) and electrolytes [28,29]. Maximum oilseed production area is centered in the North–West agro-climatic zone, where the majority of soil and groundwater sources are highly saline (1.6–17%) and have sodicity problems [30], which reduce its growth and grain yield.

Incorporating PGPR for salt tolerance and exploring the potential mechanism involved through the study of various physiological and biochemical parameters would provide a new insight into the mechanistic approach. In this research, we took mustard cultivar and exposed them to 100 mM NaCl stress. Further, these salt-stressed plants were treated with two different strains of PGPR either singly or in combination. Among the different PGPR that are widely reported for growth [31], we took *Azotobacter*, and *Pseudomonas* for our experimental work. Both these PGPR are salt-tolerant and we tested their salt tolerance before proceeding for our experiments. In addition, these salt-tolerant PGPR were synergistic in their action and in the presence of salt, they did not inhibit each other’s growth. The synergism was also tested before experimentation. On the basis of their salt-tolerant and growth-promoting activity, we studied the impact of *Pseudomonas* and *Azotobacter* either singly or in combination on salt-stressed plants. The impact of these two PGPR under salt stress was studied on total glucosinolate content (TGC), total flavonoid content (TFC), total phenolic content (TPC), carotenoid, osmolytes, ethylene evolution, antioxidative enzymes and GSH content, together with photosynthesis and growth.

## 2. Materials and Methods

### 2.1. Plant Material and Growth Conditions

Mustard (*Brassica juncea* L. var. Pusa Jagannath VSL-5) seeds were obtained from the Indian Agricultural Research Institute (IARI), New Delhi. Healthy seeds similar in shape and size were surface-sterilized with 0.01% HgCl_2,_ followed by rinsing four times with double-distilled water. Washed seeds were sown into earthen pots containing 8 kg of soil (alluvial sandy clay loam, sand 660 g kg^−1^, silt 188 g kg^−1^, clay 140 g kg^−1^, organic carbon 1.64 g kg^−1^, available N 0.41 g kg^−1^, Nitrate-N 17.21 mg kg^−1^, available P 0.08 g kg^−1^, available K 0.27 g kg^−1^, pH 7.23, soil organic carbon is 1.63 g kg^−1^, cation exchange capacity is 20.1 cmol kg^−1^) containing 8 kg of soil during the spring season of 2021 at the herbal garden of Jamia Hamdard, New Delhi, India (extending from 28.51344 N and 77.2475 E at an elevation of 782 feet above sea level). According to Köppen’s classification, the climate was humid continental (Dfb), with an average temperature of 25 °C and mean annual precipitation of 990.9 mm, mostly falling from June to September. The experiment’s design used a randomized block layout. Using the recommended basal dosages of N, P, and K for 120 kg ha^−1^, 80 kg ha^−1^, and 60 kg/ha, respectively, the soil used in pots was uniformly blended with NPK at the time of pre-sowing and 15 days after the emergence of leaves. Urea, potash, and single superphosphate (SSP) were used as the sources of N, P and, K.

### 2.2. Procurement of Bacterial Strains

Plant-growth-promoting rhizobacteria (PGPR); *Pseudomonas fluorescens* (NAIMCC-B-00340) and *Azotobacter chroococcum* Beijerinck 1901 (accession No. MCC 2351) were obtained from the National Bureau of Agriculturally Important Microorganisms (NBAIM) Mau, and National Centre for Cell Science (NCCS), Pune, respectively. The selection of these strains was based on their growth-promoting properties. A nutrient broth was used for making an overnight culture of these strains.

### 2.3. Inoculation of PGPR, NaCl Treatment and Experimental Design

Healthy and sterilized seeds were dipped in two strains, *Pseudomonas fluorescens*, *Azotobacter chroococcum,* and a combination of both strains for two hours using 1% guar gum powder as adhesive to deliver roughly 10^8^ cells per seed. In addition, the uninoculated seeds were just immersed in nutrient broth serving as control. The inoculated and uninoculated seeds (25 seeds/pot) were sown in earthen pots. After the emergence of leaves, salt stress (100 mM) was given with 600 mL per pot. Thereby, the experiment comprised eight treatments with three replicates each: T1 (control, with nutrient broth but without salt and PGPR), T2 (100 mM salt), T3 (*P*. *fluorescens*), T4 (*A*. *chroococcum*), T5 (*P*. *fluorescens* + *A*. *chroococcum*), T6 (100 mM salt + *P*. *fluorescens*), T7 (100 mM salt + *A*. *chroococcum*), and T8 (100 mM salt + *P. fluorescens* + *A*. *chroococcum*).

### 2.4. Study of Plant-Growth-Promoting Attributes of Bacterial Strains

#### 2.4.1. Indole Acetic Acid (IAA), ACC Deaminase and Siderophore Production

Indole acetic acid production was estimated using the method of [32]. Pink colour development indicated IAA production and measured its concentration at 530 nm. Pure IAA (Himedia, India) was used as the standard curve (10–100 µg mL^−1^). ACC deaminase was quantified by [33] with slight modifications. Breakdown of ACC produces α –ketobutyrate and was measured at 540 nm by comparing against the standard curve of pure α –ketobutyrate. Siderophore production was estimated by applying the method of [34], where chrome azurol S (CAS) agar plates were prepared and bacterial strains were spotted. Siderophore production was confirmed by the appearance of a yellow-to-orange-coloured halo around the bacterial growth.

#### 2.4.2. Ammonia Production, HCN Production and Phosphate Solubilization

Ammonia production (NH_3_) was measured using the method described in [35]. Brown to yellow colour development is considered a positive test for ammonia production. Production of HCN was detected using the method established by Bakker and Schipper [36], where King’s B Agar plates were prepared and the development of yellow colour is an indication of Ammonia production. P-solubilisation using both the strains was determined through the method of Pikovskaya [37] and Sindhu [38], using the spot test method on Pikovskaya medium plates. Solubilization zone formation around the bacterial colony indicated the presence of phosphate solubilization.

### 2.5. Analysis of Morphological Parameters

Biological samples from all treatments were taken randomly at 30 days after sowing (DAS) to measure root–shoot length, plant height, fresh and dry weight of root and shoot, number of leaves, leaf area. The plants were uprooted gently and the root–shoot length was measured with a metric scale and vernier calliper. The fresh weights of root and shoot were recorded utilizing an electric analytical balance (Vibra DJ 1505/Denver Instrument, APX 200). The samples were then kept in an oven (Scientific Systems 1.01) at 65 °C for 72 h to dry out before their constant weight and dry weights were calculated using an electronic balance (Denver Instrument, APX 200).

### 2.6. Biochemical Parameters

#### 2.6.1. Determination of Photosynthetic Parameters and Soluble Protein

Chlorophyll a, b and total chlorophyll were measured in fresh leaves by adopting the Hiscox and Israelstam [39] method and recording absorbance at 645, 663 using a UV–vis spectrophotometer (Model 119, Systronics, India). The chlorophyll content was expressed as mg g^−1^ FW.

Chlorophyll a (mg g^−1^ FW) = [12.7 (OD 663) − 2.69 (OD 645)] × V/1000 × W

Chlorophyll b (mg g^−1^ FW) = [22.9 (OD 663) − 4.68 (OD 645)] × V/1000 × W

Total chlorophyll (mg g^−1^ FW) = [20.2 (OD 645) + 8.02 (OD 663)] × V/1000 × W

where V, volume and W, weight. The soluble protein was measured in fresh leaves using Bradford’s method [40]. Using the bovine serum albumin (Sigma-Aldrich) as a standard and taking the absorbance at 595 nm, the amount of soluble protein was calculated and expressed as mg g^−1^ FW.

Plants’ net photosynthesis, stomatal conductance and intercellular CO_2_ were measured with infrared gas analyser (IRGA, LICOR-6400XT) in proper expanded leaves under proper bright sunlight. During the measurement, photosynthetically active radiation (PAR) was 780 µmol m^−2^ s^−1^ and atmospheric CO_2_ concentrations was 390 ± 5 µmol mol^−1^ (i.e., at light-saturating intensity).

#### 2.6.2. Estimation of Proline, Total Soluble Sugar and Glycinebetaine

Proline was estimated by applying the method described by Bates et al. [41]. L-proline (Sigma Aldrich, India) was used as a standard and absorbance was taken at 520 nm and expressed as mg g^−1^ FW. Details are provided in the Supplementary File. Total soluble sugar (TSS) was estimated using Dey’s method [42]. D-Glucose (Sigma-Aldrich) was used as a standard and absorbance was taken at 485 nm and expressed as mg g^−1^ FW. Details of the method can be obtained from the Supplementary File.

Glycine betaine (GB) content was estimated using Grieve and Grattan’s [43] method and absorbance was recorded at 365 nm.

#### 2.6.3. Estimation of Phenol, Flavonoid, Carotenoids and Glucosinolate Content

Total phenolic content (TPC) was measured by Ainsworth and Gillespie [44] using Folin–Ciocalteu (F-C) reagent. A standard curve was prepared by taking different concentrations of Gallic acid. The prepared extract was used for absorbance at 765 nm and phenolic content was expressed as mg GAE g^−1^ FW.

Total flavonoid content (TFC) was estimated via aluminium trichloride complex forming assay. A standard curve was prepared using different concentration of Rutin (10–100 µg mL^−1^); absorbance was taken at 510 nm and expressed as mg rutin g^−1^ FW.

The Hiscox and Israelstam [39] method was used to quantify the carotenoid content in fresh leaves. It measured absorbance at 480 and 510 nm and presented the results as mg g^−1^ FW. Details of the methods can be obtained from the Supplementary File.

Total glucosinolate content was estimated using previously published protocols with slight modifications [45,46]. Fresh leaf samples (1 g) were weighed and macerated in 5 mL of (70:30) methanol:water. To stop the activity of myrosinase, the extract was then heated on a water bath for 25 min at 70 °C. The extract was transferred into falcon tubes followed by centrifugation at 8000 rpm for 15 min. The collected supernatant containing total glucosinolate was then evaporated to dryness at 500 °C in a rotavapour. To 100 µL of methanolic extract, 0.3 mL of double-distilled water and 3 mL of 2 mM sodium tetrachloropalladate was added. The mixture was left for incubation for 60 min at 25 ± 50 °C. The extract was taken for absorbance at 425 nm and expressed as µmole g^−1^ FW.

#### 2.6.4. Estimation of Antioxidant Enzymes

##### Enzyme Extraction

Fresh leaves weighing 0.5 g were homogenized in 50 mM sodium phosphate buffer (pH 7.0) containing 1 mM ethylenediaminetetraacetic acid (EDTA) and 2% PVPP (polyvinylpolypyrrolidone) using a pre-chilled mortar and pestle. This homogenate was centrifuged at 13,000× *g* at 4 °C for 20 min and the supernatant collected was carried out for antioxidant enzyme assays: SOD, CAT, APX and GR.

##### Antioxidative Enzymes

Superoxide dismutase (SOD) activity was measured by Beuchamp and Fridovich [47]. Enzyme activity was measured at 560 nm and expressed as U mg^−1^ Protein min^−1^.

Catalase (CAT) activity was estimated by Beers and Sizeir [48]. A total of 3 mL catalase reaction mixture containing (100 mM phosphate buffer (pH 7.0), 0.1 mM EDTA, 20 mM H_2_O_2_) was mixed with 50 µL of enzyme extract. Monitor the decrease in the absorbance at 240 nm and quantify according to its extinction coefficient of 0.036 mM^−1^ cm^−1^ and expressed as U mg^−1^ Protein min^−1^.

Ascorbate peroxidase (APX) activity was determined by Nakano and Asada [49] (1981). First, 3 mL of assay mixture consisting (50 Mm phosphate buffer (pH 7.0), 500 mM ascorbic acid, 1 mM H_2_O_2_) was mixed with 100 µL enzyme extract. Then, APX activity was measured as decrease in absorbance at 290 nm and expressed as U mg^−1^ Protein min^−1^.

Glutathione reductase (GR) activity was assayed by Jablonski and Anderson [50] method. To 3 mL of reaction mixture comprising 100 mM phosphate buffer (pH 7.5), 1 mM oxidized glutathione, 1 mM EDTA, and 0.1 mM NADPH, 50 µLl enzyme extract was added. Oxidation of NADPH was followed by monitoring the decrease in absorbance per min at 340 nm. Enzyme activity was expressed as U mg^−1^ Protein min^−1^.

##### Content of Reduced Glutathione

The content of GSH in leaves was determined using the methods of Anderson [51]. The absorbance was recorded at 412 nm after 2 min of incubation. The Supplementary File contains details.

### 2.7. Determination of Lipid Peroxidation and H_2_O_2_ Content and Ethylene Evolution

MDA content (measure of lipid peroxidation) was determined using the method provided by Zhou and Leul [52] and absorbance was recorded at 532 nm, 450 nm, and 600 nm. Hydrogen peroxide content was measured by Velikova et al. [53]. Fresh leaves (0.5 g) were homogenized in 5 mL of 0.1% TCA in a pre-chilled mortar and pestle followed by centrifugation at 12,000× *g* for 15 min. To 0.5 mL of supernatant, 0.5 mL of 10 mM potassium phosphate buffer (pH 7.0) and 1 mL of 1 M potassium iodide (KI) solution were added. H_2_O_2_ content was measured at 390 nm.

Ethylene evolution was measured using a gas chromatograph. The detailed procedure is described in Sehar et al.’s work [54].

### 2.8. Cellular Damage Detection and Viability Measurement in Mustard Roots Using Confocal Laser Scanning Microscopy

Confocal laser scanning microscopy (CLSM) for salt and PGPR-treated roots was performed to check membrane damage and cell viability. Fresh roots were cut into small pieces, followed by dual staining with 30 μM propidium iodide (PI; HiMedia, India) and 10 μM acridine orange (AO; HiMedia, India) solution for 15 min. After being treated with a dye mixture, mustard roots were then rinsed with phosphate buffer (0.1 M, pH 7.0), and finally mounted on glass slides. Stained roots were inspected for both live and dead tissues under confocal laser scanning microscope Leica Microsystems TCS-SP5. Dead and live tissues showed PI and AO staining, respectively. Control roots were used for assessing the difference between treated and untreated roots.

### 2.9. Determination of Visible Leaf Damage via Superoxide through Histochemical Staining

Histochemical staining of superoxide ion (O_2_^−^) was performed in the leaves of the mustard plant using the method established by Kumar et al. [55] with a few modifications. To stain leaves, nitro blue tetrazolium (NBT) was used. A freshly prepared NBT solution was used for detecting (O_2_^−^). A total of 0.2 g of NBT was dissolved in 100 mL of sodium phosphate buffer to prepare the NBT solution (50 Mm, pH 7.5). Leaf samples were then immersed in NBT solution and incubated for an overnight period at room temperature. The samples were then boiled in absolute ethanol for 20 min and photographs were then taken.

### 2.10. Compatibility Assay

A compatibility assay was performed using the well diffusion method. To check their compatibility under in vitro conditions, 50 µL culture of *P*. *fluorescens* was spread on a nutrient agar (NA) plate and 50 µL culture of *A*. *chroococcum* was filled in the well formed on the plate; later, the NA plate was kept at 28 ± 2 °C for 24 h in an incubator. Presence or absence of an inhibition zone around the well would confirm the antagonism and synergism, respectively, between two PGPR.

### 2.11. Salt Tolerance Assay for PGPR Strains

Rhizobacterial strains, i.e., *Pseudomonas fluorescens* (NAIMCC-B-00340) and *Azotobacter chroococcum* Beijerinck 1901 (MCC 2351), were characterized for salinity tolerance. The ability of both strains to tolerate 100 mM salt concentration was evaluated by growing (streaking) both strains on a nutrient agar (NA) plate amended with 0 mM, 50 mM and 100 mM salt concentration. For this purpose, nutrient agar containing the desired amount of NaCl was autoclaved and poured into plates. After solidifying the plates, both strains were streaked on plates to check their growth under salt conditions.

### 2.12. Statistical Analysis

Data collected from the completely randomized block design experiments were analysed statistically using analysis of variance (ANOVA) by SPSS 17.0 for windows and presented as mean ± SE (*n* = 4). The least significant difference was calculated for the significant data at *p* < 0.05. Bars with the same letter were not significantly different using the least significant difference (LSD) test at *p* < 0.05. The PCA and Pearson correlation were carried out using OriginPro software. To create biplots, the first two components (PC1 and PC2) showing the maximum variance in the datasets were considered.

## 3. Results

### 3.1. Properties of Pseudomonas fluorescens and Azotobacter chroococcum

**Figure 1:**
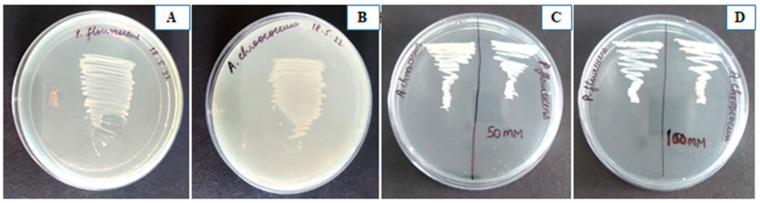
(**A**,**B**) are *Pseudomonas fluorescens* and *Azotobacter chroococcum*, respectively, streaked on 0 mM NaCl NA plate, (**C**) is *Pseudomonas fluorescens* and *Azotobacter chroococcum* streaked on 50 mM NaCl nutrient agar plate and (**D**) on 100 mM nutrient agar plate.

Both these strains were Gram-negative, produced indole acetic acid (IAA), ACC deaminase, siderophore, ammonia, HCN and phosphate solubilisation, as shown in Table 1. They were salt-tolerant at 100 mM NaCl concentration, as shown in Figure 1.

### 3.2. Application of PGPR Strains alleviated Growth Characteristics under Salt Stress

Growth characteristics, such as root length, shoot length, plant height, root–shoot biomass, number of leaves and leaf area, were significantly decreased with the salinity (Table 2). This inhibiting effect of salinity may result from a variety of physiological mechanisms, including a decline in meristematic activity and/or cell expansion. The data revealed that all the attributes declined with the salt stress. Contrary to this, application of PGPR strains significantly enhanced growth parameters, thus mitigating the effects of salt stress. Both PGPR strains alleviated plant growth in control as well as stressed conditions; however, the trend observed was maximum in *Pseudomonas* + *Azotobacter*, followed by *Pseudomonas*, and then in *Azotobacter*. Salt-stressed plants inoculated with *Pseudomonas*, *Azotobacter* and *Pseudomonas* + *Azotobacter* enhanced root length (RL) by 47.8, 42.5 and 78.4%; shoot length (SL) by 21.61,16.08 and 38.2%; height of plant by 30.7, 25.2 and 52.1%; leaf number by 63.6, 54.5 and 109%; and leaf area by 33.3, 25.8 and 46.7%, respectively, in comparison to control.

### 3.3. Impact of PGPR on Photosynthetic Pigments

**Figure 2:**
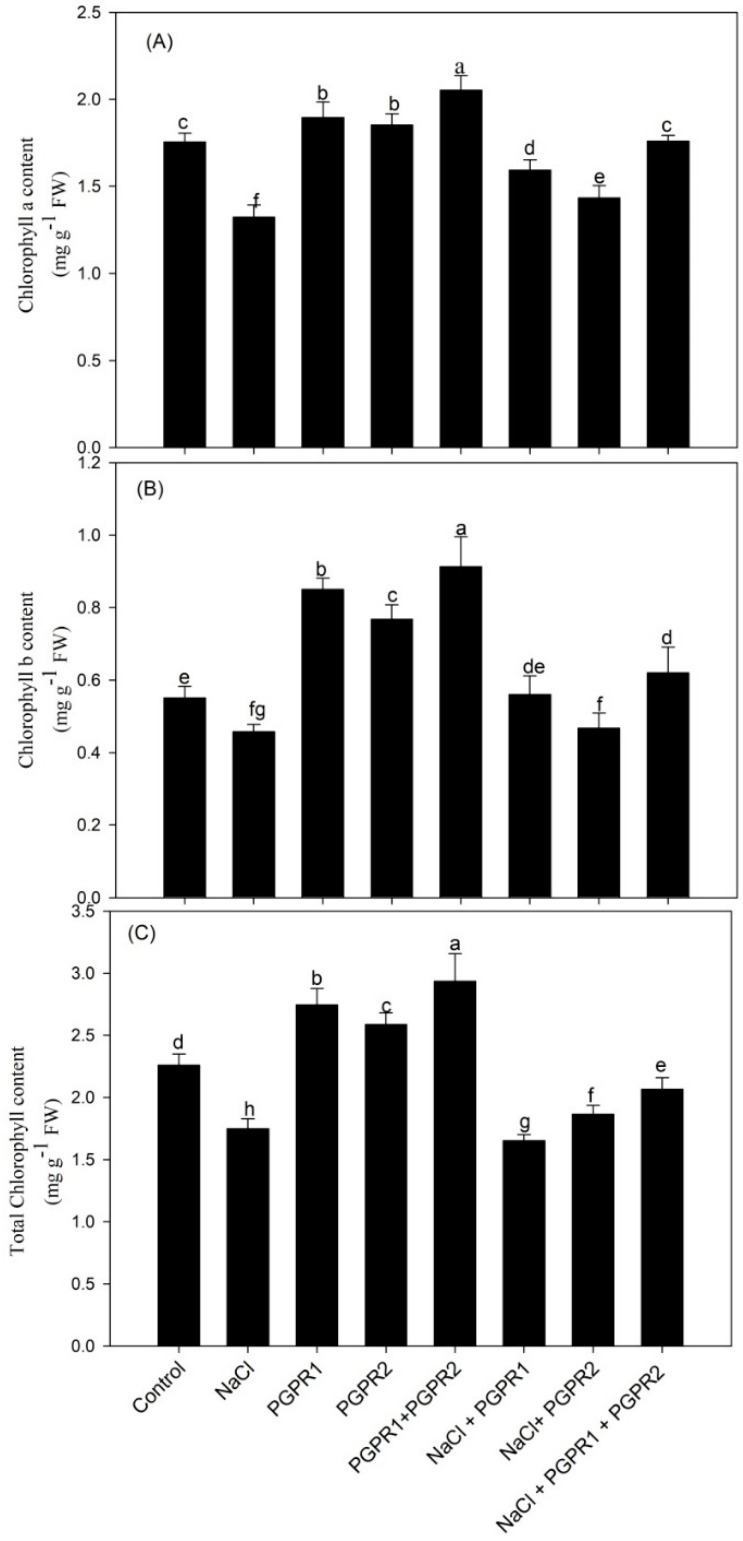
Effect on chlorophyll a (**A**), chlorophyll b (**B**), and total chlorophyll (**C**) in mustard cultivar Pusa Jagannath exposed to control or salt stress with or without *Pseudomonas fluorescens* (PGPR1) or *Azotobacter chroococcum* (PGPR2) either singly or in combination. Data are presented as treatment mean ± SE (*n* = 4). Data followed by same letter are not significantly different using LSD test at *p* < 0.05.

Salt stress significantly decreased pigment content (chlorophyll a, b, total chlorophyll) and protein content. However, inoculation with two bacterial strains (*P. fluorescens*, *A. chroococcum* and combination of two) reduced the harmful effects of NaCl and increased the evaluated parameters. In salt-stressed plants with Chl a content, 1.323 mg g^−1^ FW was increased to 1.492, 1.434 and 1.555 mg g^−1^ FW when inoculated with *Pseudomonas*, *Azotobacter* and *Pseudomonas* + *Azotobacter*, respectively, in comparison to control. The same trend was observed in Chl b, total Chl and protein content; Chl b shot up from 0.458 mg g^−1^ FW to 0.489, 0.468 and 0.548 mg g^−1^ FW; total Chl surged from 1.749 mg g^−1^ FW to 1.943, 1.865 and 2.066, respectively. The highest values for Chl a, b and total chlorophyll content were found in the plants inoculated with the combination of two strains which increased by 17.5, 19.6 and 18.1%, respectively, over the course of salt-stressed plants’ (T2) treatment (Figure 2).

### 3.4. Gas Exchange Parameters

**Figure 3:**
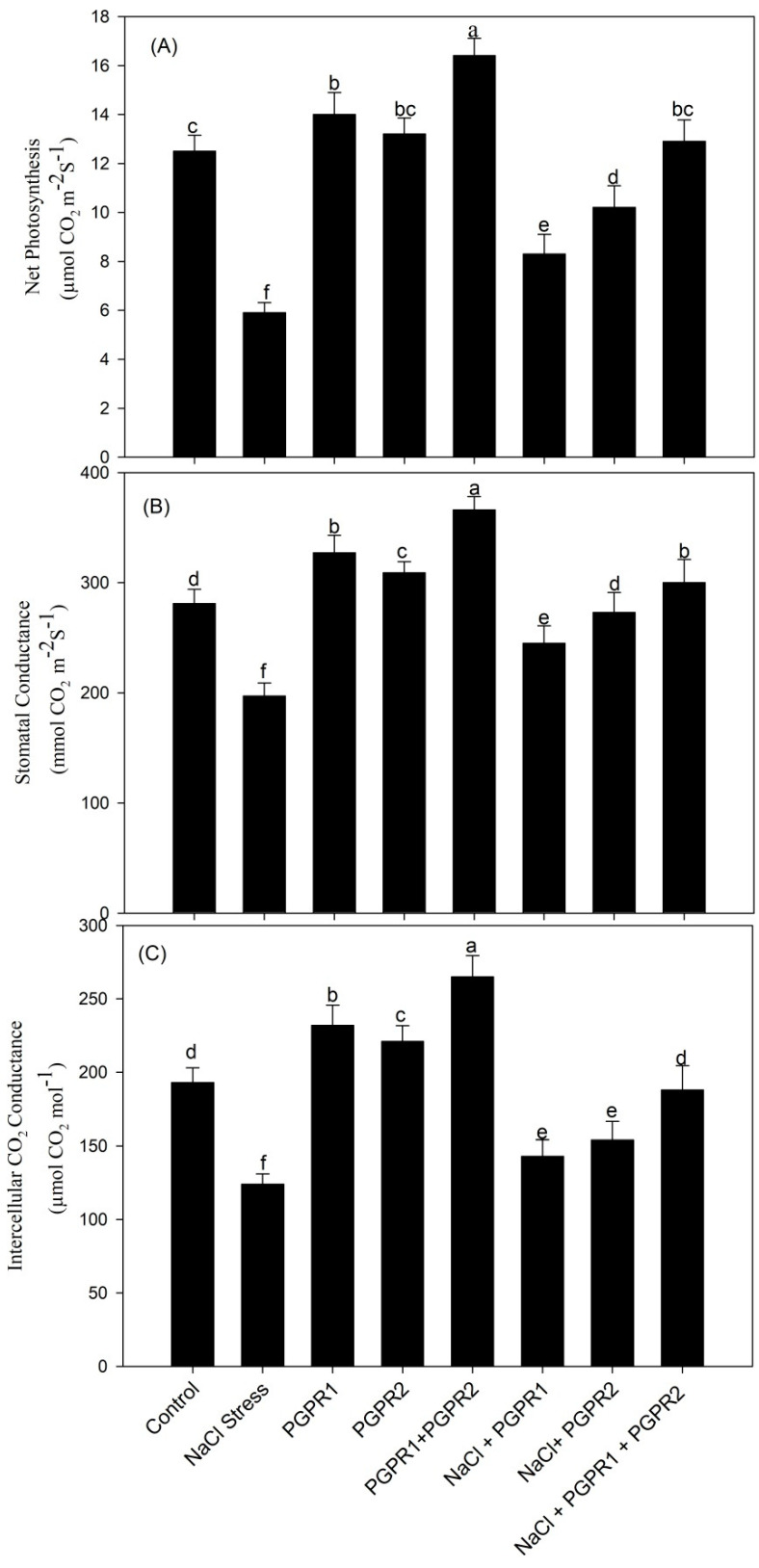
Effect on net photosynthesis (**A**), stomatal conductance (**B**), and intercellular CO_2_ concentration (**C**) in mustard cultivar Pusa Jagannath exposed to control or salt stress with or without *Pseudomonas fluorescens* (PGPR1) or *Azotobacter chroococcum* (PGPR2) either singly or in combination. Data are presented as treatment mean ± SE (*n* = 4). Data followed by same letter are not significantly different using LSD test at *p* < 0.05.

Gas exchange parameters (net photosynthesis (PN), stomatal conductance (Gs) and intercellular CO_2_ (Ci) expressed considerable decline under salt stress. Studied parameters PN, Gs and Ci showed a decrease of 2.1-, 1.4- and 1.5-fold, respectively, when compared with the control. Bacterial inoculation enhanced gas exchange parameters in treated and as well as untreated plants. Considering the comparative performance of bacterial strains, a mixture of *Pseudomonas* + *Azotobacter* proved best for the parameters studied, whereas *Azotobacter* showed minimal increase. With respect to salt treatment (T2), *Pseudomonas* + *Azotobacter* improved PN, Gs and Ci by 0.5-, 0.6- and 0.6-fold, respectively (Figure 3).

### 3.5. Osmolytes Accumulation Increased under Salt Stress and Helped in Stress Tolerance

Bacterial strains increased the accumulation of proline and glycine betaine under control and stressed conditions. Salt stress markedly enhanced proline and glycine betaine content by 2- and 1.4-fold, respectively, over control plants. Inoculation of salt-stressed plants with *Pseudomonas* significantly enhanced proline and glycine betaine content by 38.8% and 26.3%, respectively, over salt treatment (T2). *Azotobacter* inoculation increased proline and GB content by 31.4 and 19%, respectively, when compared to T2 treatment. In contrast, the combination of both *Pseudomonas* and *Azotobacter* boosted proline and GB content maximum by 60.3 and 32.4%, respectively, with respect to salt-stressed plants.

**Figure 4:**
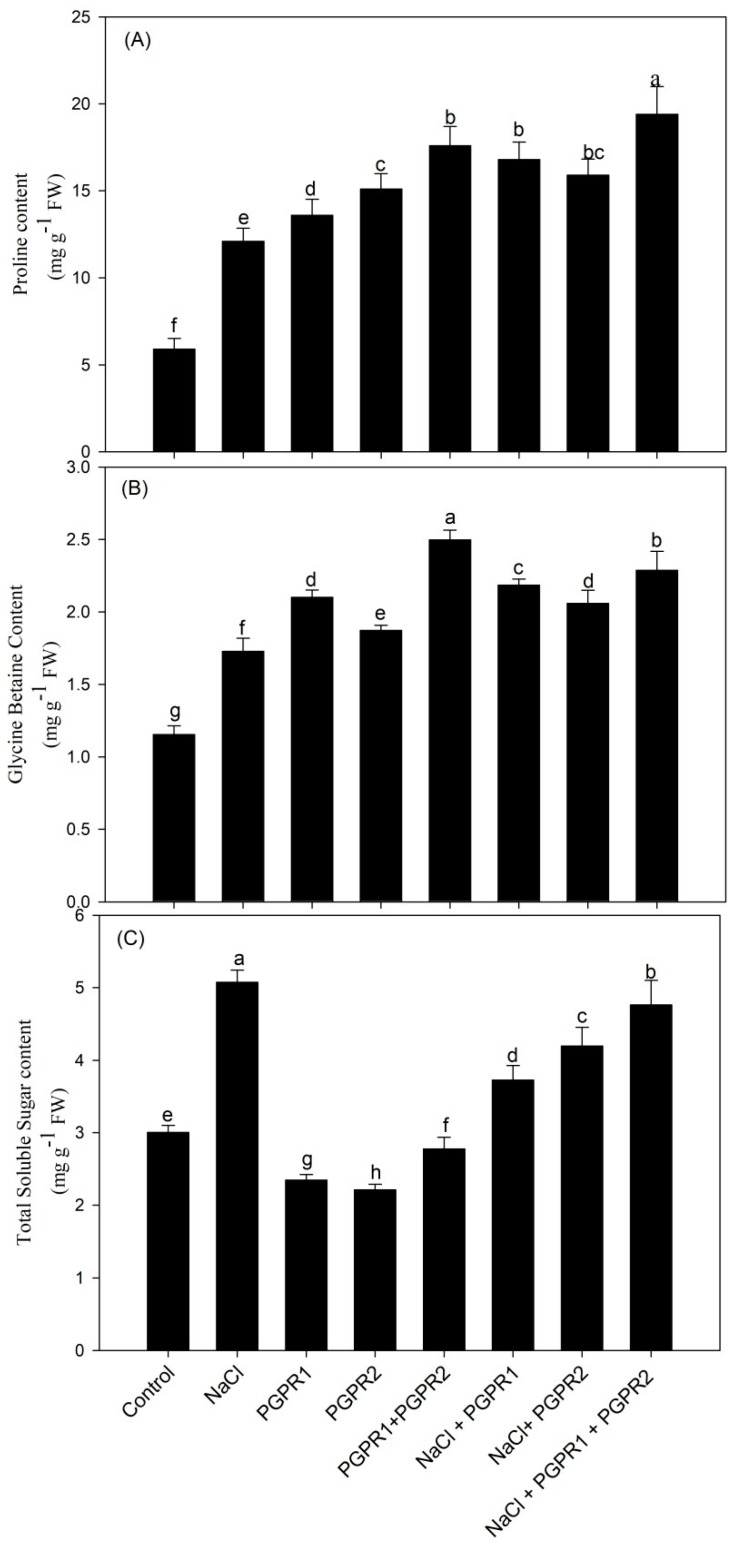
Effect on proline content (**A**), glycine betaine content (**B**), and total soluble sugar content (**C**) in mustard cultivar Pusa Jagannath exposed to control or salt stress with or without *Pseudomonas fluorescens* (PGPR1) or *Azotobacter chroococcum* (PGPR2) either singly or in combination. Data are presented as treatment mean ± SE (*n* = 4). Data followed by same letter are not significantly different using LSD test at *p* < 0.05.

A pronounced increase in total soluble sugar content was noted under salt treatment. In contrast to the control, sugar content increased by 68.9% under salt stress. Application of bacterial strains, however, resulted in sugar decline in both stressed as well as unstressed conditions. Plants exposed to salt that were inoculated with *Pseudomonas*, *Azotobacter*, and *Pseudomonas* + *Azotobacter* revealed reductions in sugar content of 26.5, 17.3, and 6.1%, respectively (Figure 4).

### 3.6. Impact on Total Phenol, Total Flavonoid, Carotenoids and Total Glucosinolate Content

The control plants supplemented with bacterial strains exhibited an increase in phenol and flavonoid content over the control plants. Salt (100 mM) application also enhanced phenol and flavonoid content by 44.4% and 48.3%, respectively, over control plants. Salt-treated plants inoculated with *Pseudomonas*, *Azotobacter* and *Pseudomonas* + *Azotobacter* amplified phenol and flavonoid content by 15.4, 10.2, 26.7% and 11.6, 9.5, 17.5%, respectively, when compared to T2 treatment.

A considerable decrease in carotenoid content was recorded in salt-stressed samples with respect to control. Application of bacterial strains increased their carotenoid content both in control as well as stressed plants. Maximum increase was observed in *Pseudomonas*+ *Azotobacter*, followed by *Pseudomonas*, and then *Azotobacter* in both stressed and unstressed plants.

The amount of total glucosinolate increased in all of the treatments compared to control T1, but salt stress had the highest amount at 57.4%. Bacterial strains increased the amount of glucosinolate in control plants while lowering it in salt-stressed plants. In control plants, *Pseudomonas*, *Azotobacter* and *Pseudomonas* + *Azotobacter* enhanced glucosinolate content by 27.8, 23.7 and 45.4%, respectively. In contrast, in the salt-stressed plants, glucosinolate content decreased by 15.2, 20 and 4.5%, respectively (Table 3).

### 3.7. Impact of PGPR on Lipid Peroxidation and H_2_O_2_ Content and Ethylene Evolution

**Figure 5:**
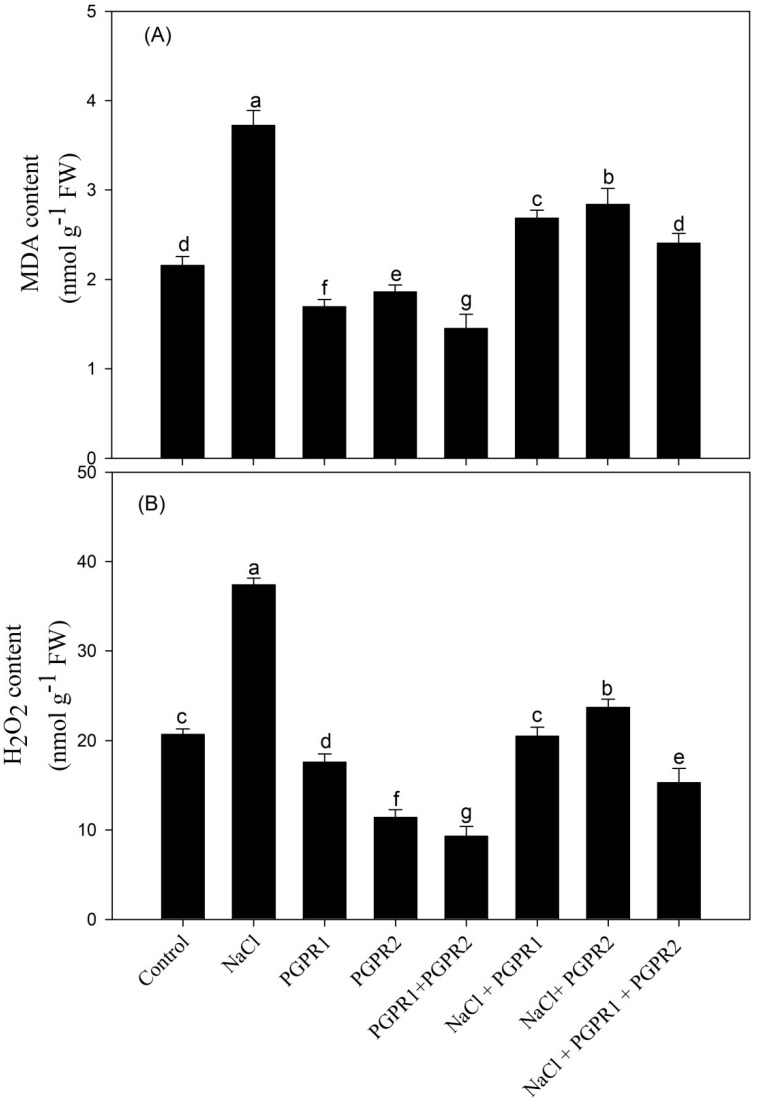
Effect on MDA (**A**) and H_2_O_2_ (**B**) content in mustard cultivar Pusa Jagannath exposed to control or salt stress with or without *Pseudomonas fluorescens* (PGPR1) or *Azotobacter chroococcum* (PGPR2) either singly or in combination. Data are presented as treatment mean ± SE (*n* = 4). Data followed by same letter are not significantly different using LSD test at *p* < 0.05.

Lipid peroxidation and H_2_O_2_ content are important indicators of oxidative stress. The mustard plants under salinity stress (100 mM) showed a remarkable increase in MDA and H_2_O_2_ content by 1.7- and 1.8-fold, respectively. On the other hand, bacterial inoculation under saline and non-saline treatments considerably reduced its content. Maximum reduction in MDA and H_2_O_2_ content was observed in the plants inoculated with the combination of *Pseudomonas* + *Azotobacter*, by 35.3 and 59.0%, respectively, in comparison with the salt treatment (T2) (Figure 5).

**Figure 6:**
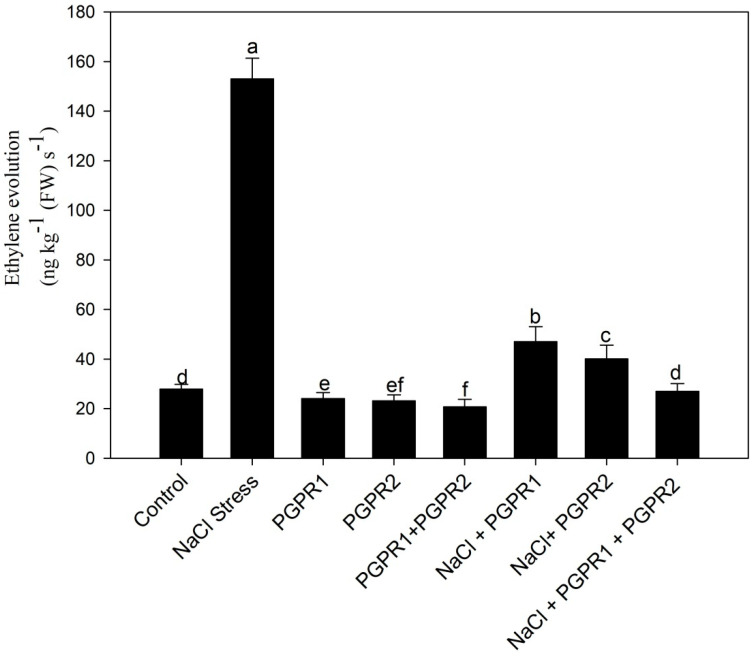
Effect on ethylene evolution in mustard cultivar Pusa Jagannath exposed to control or salt stress with or without *Pseudomonas fluorescens* (PGPR1) or *Azotobacter chroococcum* (PGPR2) either singly or in combination. Data are presented as treatment mean ± SE (*n* = 4). Data followed by same letter are not significantly different using LSD test at *p* < 0.05.

Ethylene content was maximum under salt stress and decreased with the application of PGPR. Maximum reduction in ethylene content was observed with combined PGPR application (Figure 6).

### 3.8. Alleviating Effect of PGPR on Antioxidative Enzymes and GSH

**Figure 7:**
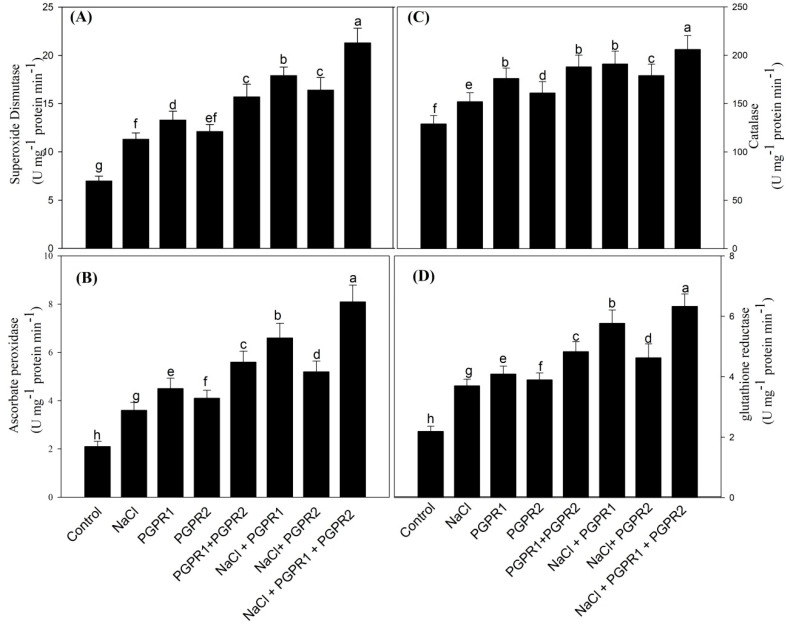
Effect on SOD (**A**), CAT (**B**), APX (**C**) and GR (**D**) activity in mustard cultivar Pusa Jagannath exposed to control or salt stress with or without *Pseudomonas fluorescens* (PGPR1) or *Azotobacter chroococcum* (PGPR2) either singly or in combination. Data are presented as treatment mean ± SE (*n* = 4). Data followed by same letter are not significantly different using LSD test at *p* < 0.05.

Antioxidant activities in mustard plants were higher under salinity stress than they were under normal growth conditions. The content of antioxidative enzymes (SOD, CAT, APX, and GR) enhanced by 61.6, 17.8, 68.1 and 62.4%, respectively, with respect to control plants. However, inoculation of plants with bacterial strains increased antioxidants’ activity in salt-stressed as well as unstressed plants. Interestingly, among bacterial strains (*Pseudomonas*, *Azotobacter* and *Pseudomonas* + *Azotobacter*), the combination of *Pseudomonas* + *Azotobacter* maximized the content of SOD, CAT, APX, and GR by 0.5-, 0.7-, 0.4- and 0.5-fold, respectively, over salt-treated plants (T2) (Figure 7).

**Figure 8:**
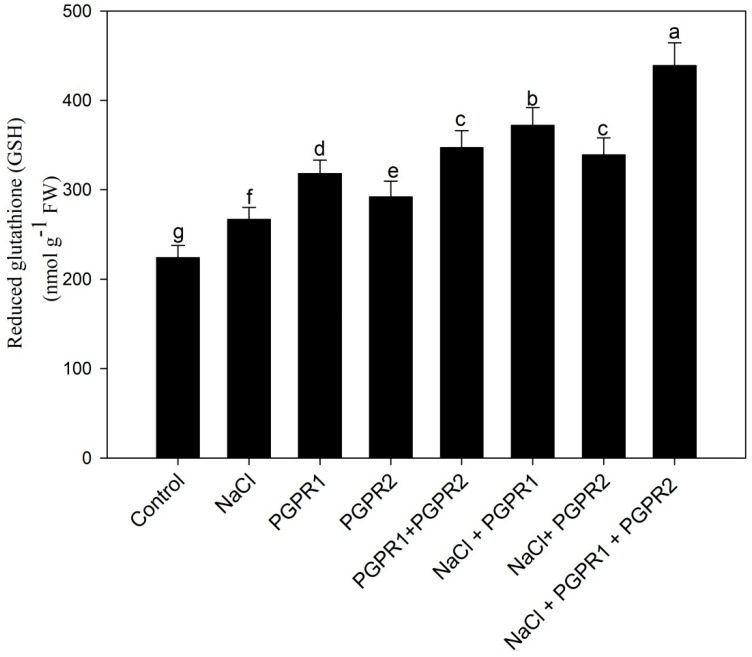
Effect on reduced GSH content in mustard cultivar Pusa Jagannath exposed to control or salt stress with or without *Pseudomonas fluorescens* (PGPR1) or *Azotobacter chroococcum* (PGPR2) either singly or in combination. Data are presented as treatment mean ± SE (*n* = 4). Data followed by same letter are not significantly different using LSD test at *p* < 0.05.

GSH content was increased under salt stress but supplementation of PGPR further increased GSH content and, when in combination, it was maximal (Figure 8).

### 3.9. Cellular Damage to Mustard Roots under Salt Stress as Determined via CLSM and Overcoming the Damage Using PGPR

**Figure 9:**
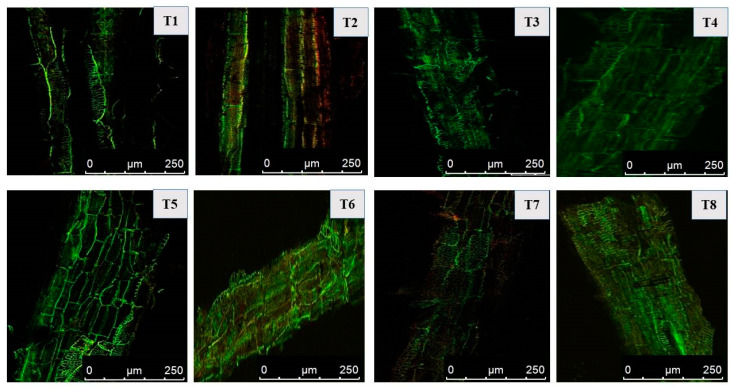
Effect on cellular damage to mustard roots in mustard cultivar Pusa Jagannath exposed to control or salt stress with or without *Pseudomonas fluorescens* (PGPR1) or *Azotobacter chroococcum* (PGPR2) either singly or in combination.

Roots of mustard plants treated with 100 mM NaCl experienced oxidative stress leading to tissue death. When plants were subjected to salinity, the increased PI red fluorescence from the mustard roots cells indicated cell death owing to cellular membrane breakdown. PI dye (a DNA intercalating dye) only penetrates the membranes of dead cells; hence, the cells accumulate the red colour and due to this reason, the PI dye is considered a good indicator for stressed conditions. However, roots inoculated with PGPR displayed less cell membrane damage than un-inoculated roots. Roots inoculated with *Pseudomonas* + *Azotobacter* accumulated minimal red colour (PI dye) in stressed as well as unstressed conditions. In contrast to this, the roots of untreated plants showed strong green fluorescence from (AO) acridine orange and much less PI fluorescence, indicating limited cellular damage (Figure 9).

### 3.10. Visible Damage Due to Superoxide Formation in Salt-Stressed Leaves and Its Alleviation Using PGPR

**Figure 10:**
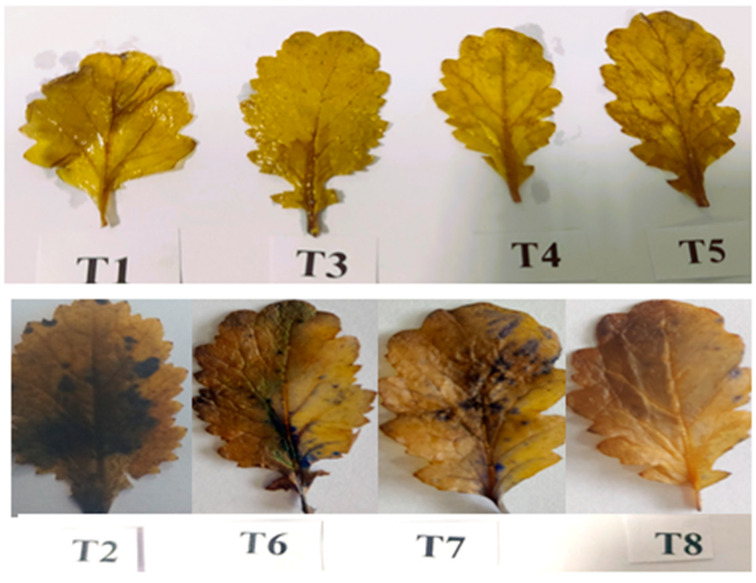
Effect on visual damage to mustard leaves due to superoxide formation in mustard cultivar Pusa Jagannath exposed to control or salt stress with or without *Pseudomonas fluorescens* (PGPR1) or *Azotobacter chroococcum* (PGPR2) either singly or in combination.

Histochemical staining was conducted to determine in situ accumulation of (O_2_^−^), which represents damage caused by ROS. On leaves of a plants treated with 100 mM NaCl (T2), more dark blue spots corresponding to O_2_^−^ were seen as NBT was converted to formazan when compared to control (T1). However, no obvious prominent dark blue spots were observed in bacterial-inoculated leaves. Rhizobacterial inoculations with *Pseudomonas*, *Azotobacter* and combination of both (*Pseudomonas* + *Azotobacter*) showed no dark blue spot as PGPR enhanced antioxidant activity (Figure 10).

### 3.11. Synergism between Two PGPR

The results of the compatibility assay indicated that both the strains could grow together since there was no inhibition zone around the well as there was no interference with one another’s ability to grow. A photo is provided in the Supplementary File.

### 3.12. Principal Component Analysis

**Figure 11:**
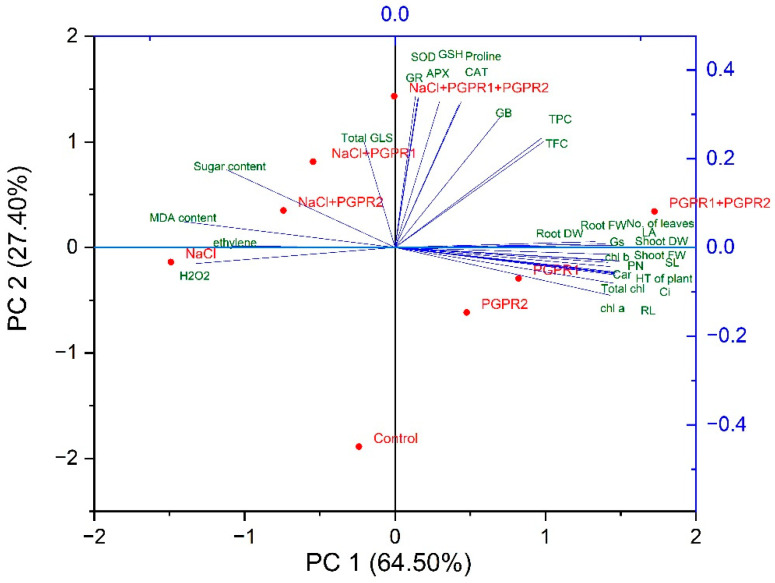
The PCA was applied to study the interaction between variables (parameters studied) and observation (treatments) with or without salt stress.

The scores of PCA to evaluate the effects of PGPR 1 and PGPR 2 on mustard plants under salt stress are presented in Figure 10. PC1 and PC2 accounted for 91.9% of the total variance in the dataset. Between them, PC1 contributed 64.5% and PC2 contributed 27.4% total variation. All the treatments were distributed successfully by the first two principal components (Figure 10). The salt stress treatment was distributed along with the oxidative stress biomarkers (H_2_O_2_ and MDA content) and ethylene content. The various parameters observed in PCA biplot were divided into three clusters. Parameters such as H_2_O_2_ and MDA content, ethylene content, and sugar content were close to those of the salt stress treatment. The parameters of growth and photosynthesis were close to those of the PGPR treatments without stress. On the other hand, antioxidants (SOD, CAT, APX, GR, GSH), proline, glycine betaine, TFC and TPC were close to the values of the combined treatment of PGPR 1 + PGPR 2 in the presence of salt stress (Figure 11). The oxidative stress biomarkers and ethylene biosynthesis showed a negative correlation with plant growth and photosynthesis parameters. From the biplot, it is clear that the antioxidants, osmolytes and secondary metabolites were in between the oxidative stress, plant growth and photosynthesis, suggesting their role in combating heat stress. Therefore, the correlation biplot portrays a close association between PGPR in the salt acclimation of mustard plants (Figure 11).

### 3.13. Pearson Correlation

**Figure 12:**
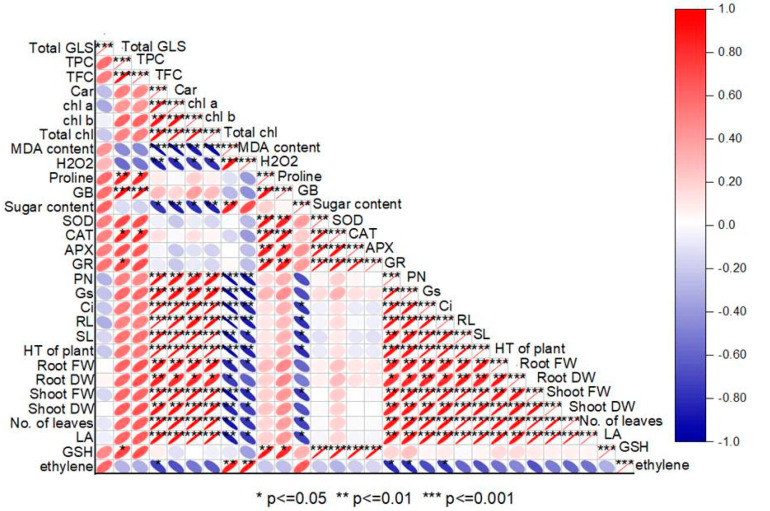
Pearson correlation heatmap was prepared to study the interacting among different studied parameters.

A Pearson correlation heatmap was drawn to study the relationship between the studied parameters (Figure 11). A significant (*p* ≤ 0.05; *p* ≤ 0.01 and *p* ≤ 0.001) positive correlation was observed among the observed attributes of growth, photosynthesis, antioxidant and secondary metabolites. Salt stress-induced oxidative stress showed a negative correlation with the plant growth and photosynthetic attributes. Ethylene production showed a strong correlation with H_2_O_2_, MDA content, showing stress-specific production of ethylene. Antioxidant enzymes (SOD, CAT, APX and GR) were negatively correlated with H_2_O_2_ and MDA content. Moreover, TPC, TFC and Car showed a positive correlation with PDM, LA, PN, gs, Ci, signifying the positive role of these metabolites in improving growth and photosynthesis under salt stress (Figure 12).

## 4. Discussion

### 4.1. Impact of Combined PGPR on Mustard Morphological Characteristics under Salt Stress

Increasing salinity stress in agricultural soil is a serious concern since it hinders plant growth and causes large yield losses [56]. However, the use of PGPR in crop plants grown in salty environments can counteract the negative effects of salinity and promote plant growth and survival [57,58,59,60,61]. In the present study, salt-stressed mustard plants inoculated with two types of rhizobacterial strains showed an increase in root length (RL), shoot length (SL), fresh weight (FW), dry weight (DW), leaf area (LA), leaf number and height when compared to un-inoculated ones. The elevated growth parameters in PGPR-treated plants could be possible due to the production of different phytohormones [62] in the rhizosphere of plants, causing cellular division and elongation [63]. PGPR causes root proliferation and increased root length through the secretion of IAA, which causes an increase in root surface area and ultimately leads to better nutrient absorption, for improved plant growth under stressed condition as reported by Yasmin et al. [64] in the case of *Pseudomonas* sp. and by Kalaiarasi and Dinakar [65] in the case of *Azotobacter*. These nutrients are crucial to the expansion of the leaf area [66,67]. In the previous studies, an increase in biomass and height was documented in rapeseed [68], soybean [69], and tomato [70] with PGPR. Both PGPR *Pseudomonas* [71] and *Azotobacter* [72] have ACC deaminase activity. Under salinity stress, ACC deaminase reduces stress-induced ethylene synthesis by converting ACC into ammonia and α ketoglutarate, promoting the development of longer root systems. It is well-documented in the previous studies that the supplementation of PGPR with ACC deaminase activity helps in reducing salinity stress [56,61,71]. Under salinity stress, PGPR regulate osmotic balance and ion homeostasis either through the modulation of phytohormone or by impacting metabolite and antioxidant activity or osmolyte accumulation to reduce osmotic stress and ion toxicity [73]. We have reported this result in light of the traits that are known for these PGPR and we also analysed these traits in Table 1. Both PGPR strains are able to produce IAA, which could be the reason for salt stress-induced growth reduction as the decrease in endogenous hormone levels was responsible for the deleterious effect of salinity on plant growth [74,75], while addition of bacterial auxins benefited plant development in environments with high salt concentrations [76,77]. PGPR releases metal-chelating substances, such as siderophores, which help in the uptake of various metals such as Zn, Fe, Cu [78,79,80]; interestingly, both bacterial strains in our study are able to produce siderophores. In addition to this, HCN production promotes plant growth and development by arresting the growth of pathogens and serving as a biocontrol agent [81]. The growth of salt-stressed plants is also affected by limited phosphate nutrition [82] and both the strains are able to solubilize phosphate, leading to its availability to plants, thereby increasing growth.

### 4.2. Impact of PGPR on Photosynthetic Traits in Mustard under Salt Stress

Photosynthesis is a fundamental plant physiological activity that maintains plant development and improves the plant’s tolerance to external challenges [83]. Overall, Chl a, b, total Chl, and carotenoids decreased with the salt stress in our study. Exposing mustard plants to salt stress leads to the induction of changes in the pigment–protein complex [84]. Elkelish et al. [85] and Ahanger et al. [86] concluded that salinity enhances chlorophyllase activity and reduces N-uptake, leaf water potential, and photosynthetic efficiency. Plants inoculated with root-colonizing rhizobacteria enhanced chlorophyll content because the iron (Fe^3+^) present in the Fe^3+^-siderophore complex on the bacterial membrane is decreased in order to make iron available to both the plant and the bacteria [87,88]. Chlorophyll-containing iron that serves as a chelating agent and PGPR make iron available to plants, increasing their photosynthetic activity and general growth. PGPR improves the solubilization and uptake of nutrients, thus modulating plant growth and development [89]. Various studies have proved that inoculation with PGPR improves chlorophyll content under salt stress [90,91]. Our results also show the enhancement of chlorophyll content with inoculation of bacterial strains under salt-stressed conditions. Inoculation with PGPR increases stomatal conductance [92] and water and ion absorption [93], which, in turn, promotes the production of photosynthetic pigments and net photosynthesis. Habib et al. [94] explained that ACC deaminase activity of PGPR improved photosynthetic efficiency by reducing ethylene biosynthesis. In addition to this, the phosphate solubilization trait of PGPR improves nutrient absorption activity, which is essential for the biosynthesis of photosynthetic pigments needed for the light harvesting complex [95].

### 4.3. PGPR in Combination Maximally Alleviated Oxidative Stress through an Increase in Antioxidative Enzymes and Antioxidant GSH

Reactive oxygen species are formed in response to salt exposure, causing extreme oxidative damage to nucleic acids, lipids and proteins [96]. Antioxidant systems have a crucial role in shielding plants from oxidative stress [97]. Superoxide dismutase, as the name suggests, causes the dismutation of superoxide anion to H_2_O_2_ [98]. CAT plays an important function in lowering the level of H_2_O_2_ in peroxisomes and detoxifying them [99], APX scavenges H_2_O_2_ by regulating their signals [100], and GR balances the high cellular glutathione/glutathione disulfide (GSH/GSSG) ratio by prompting a reduction reaction of glutathione disulfide (GSSG) to glutathione (GSH) taking part in H_2_O_2_ detoxification [101]. The PGPR-induced antioxidative enzymes are considered to be good contributor to the salt stress tolerance in plants by reducing H_2_O_2_ [102]. In our current study, the activities of antioxidant enzymes are significantly higher in PGPR-inoculated plants and maximal in combination of both *P. fluorescens* + *A. chroococcum* strains. *Pseudomonas fluorescens* strains have been found to improve the sweet corn antioxidant system against salinity stress [103]. Increased activity of antioxidant enzymes and osmoprotectants were also observed in *E. cloacae* PM23-inoculated plants under salt stress in maize compared to control plants [104]. GSH content increases with salt stress and is at its maximum in the salt plus PGPR combination. The increase in GSH under salt stress is reported in wheat [105], which was responsible for salt tolerance. The interactions of ethylene with GSH and that of S with GSH are also reported and the associated stress tolerance has been studied [54,106]. Sofy et al. [107] reported increased GSH and antioxidative enzymes’ content with endophytic bacteria producing ACC deaminase in *Pisum sativum*.

### 4.4. PGPR When Applied Together Maximally Increased Osmolytes Accumulation to Protect against Salt-Induced Osmotic Stress

The salt ions’ accumulation around the plant’s root causes osmotic stress under saline conditions, eventually leading to osmotic imbalance [108]. Accumulation of osmoprotectants such as proline, glycine betaine, and soluble sugar is an adaptive response of plants to various stresses, such as salinity, as they have a role in the osmotic adjustment and alleviate cellular oxidative damage [57,73]. A significant increase in proline and glycine betaine was observed in our present study on PGPR treatment. This may be due to the reason of synthesis and accumulation of free amino acids under stressed conditions [109]. The most prominent reason behind alleviated proline levels by PGPR may be proline-synthesizing enzymes and the downregulation of catabolizing enzymes as described by the authors of [110]. Osmoprotectants increase the resistance of plants against salt stress by stabilizing the protein conformation, cytosolic pH, balancing redox condition of cell, PSII and membrane integrity, and the activity of various enzymes [111,112]. Total soluble sugar (TSS) is an important parameter and is synthesized and accumulated in cytosol under salt stress. In our results, TSS content was higher in stressed conditions to improve osmotic adjustment and maintain turgor under salinity. Sugars have essential roles in maintaining various physiological processes such as carbohydrate metabolism, secondary metabolism, and certain other activities involving osmotic balance under stressed conditions [113]. However, supplementation of PGPR caused a decline in TSS content. This could be attributed to the better utilization of TSS by PGPR that prevented its accumulation. The increased sugar acts as an osmolyte to prevent stress-induced ionic and osmotic stress; however, PGPR application results in decreasing the stress through an increase in antioxidative enzymes that led to the scavenging of ROS and the utilization of TSS to promote growth under stress. Iqbal et al. [101] reported a decrease in glucose content with nitric oxide application under stress due to a reduction in stress. Sehar et al. [54] found that in wheat, optimum ethylene reduces glucose sensitivity by enhancing stress tolerance under salt stress; glucose was highest under stress and decreased with a reduction in stress.

### 4.5. PGPR Application Reduced Ethylene Evolution under Salt Stress and Maximally When Supplemented in Combination

Ethylene is a phytohormone that is produced endogenously by the plant for the control of plant development. When a plant is under salinity stress, it produces more ethylene, which raises its endogenous level [114]. Various PGPR with ACC deaminase activity have been shown to improve the growth of numerous crops as well as plant tolerance to various abiotic stresses by lowering stress hormone ethylene levels through a reduction in ACC content [115,116,117]. ACC played a negative role under salinity in regulating tomato seedling’s growth [118]. Lowering ethylene production under salt stress resulted in better growth in wheat [54]. In our report, inoculation of mustard seeds with bacterial strains singly or in combination both reduced ethylene levels. However, combination of both strains (*P. fluorescens* + *A. chroococcum*) maximally reduced ethylene levels. Reduced ethylene levels in both strains, *P. fluorescens* [119] and *A. chroococcum,* occurs because both strains have ACC deaminase activity, which limited the deleterious effects of ethylene on plant growth and development under salinity stress (Table 1). Salt stress increases the synthesis of ROS (H_2_O_2_ and MDA content) and ethylene as observed in this study. Both ROS and ethylene damage plants when at high level. Stress ethylene synthesis is the plant’s inherent ability to adapt to stress situations but at the expense of reduced photosynthesis and growth. In a strategy to overcome this compromise, PGPR might play a major role as they reduce ACC content through their ACC deaminase activity. Ethylene production increases under salinity stress and its signalling is required for the plant’s immediate response to salinity and adjustment in order to cope with stress. However, continuous stress leads to excessive ethylene production, which inhibits plant growth and development and eventually leads to the death of plants [120]. In rice, ethylene production increased due to salinity stress via the MAPK cascade that stabilizes ACS, which promoted ROS accumulation and growth inhibition [121,122]. Thus, excessive ethylene leads to ROS accumulation as observed in this study, which causes a deleterious effect on plant growth. Ethylene reduction using PGPR via a decrease in ACC content is, therefore, one strategy for ROS reduction and plant survival. Moreover, when ethylene is reduced to an optimum level, it enhances growth through an increased antioxidative metabolism. In fact, ethylene level is a controlling factor for growth promotion or inhibition depending on concentration [123].

### 4.6. PGPR Application Enhanced Secondary Metabolite Production but Decreased Glucosinolate Content under Salt Stress

PGPR supplementation under salt stress increased the accumulation of secondary metabolites but decreased glucosinolate content. Brassica is rich in various bioactive phytochemicals such as glucosinolates and phenolic compounds, including phenols, flavonoids, and carotenoids [124,125,126]. These phenolic compounds have a crucial role in absorbing and neutralizing free radicals, quenching singlet oxygen, and detoxifying peroxides [59,127]. The mustard variety (Pusa Jagannath) with 100 mM salt stress showed enhanced TPC and TFC for promoting defensive mechanisms and regulating the normal cellular functioning [128]. By interacting with a variety of protein kinases, such as mitogen-activated protein kinases, flavonoids are thought to play a role in signalling systems that reflect the onset of cellular differentiation and growth in plants [129]. It has been considered that flavonoids act as chelators under salinity stress [130]. Maximum elevated content of TPC and TFC was found in the combination of both strains (*P. fluorescens* + *A. chroococcum*). An increase in TPC and TFC content was also observed with the inoculation of *Pseudomonas* and *Azotobacter* in reports of Kandoliya and Vakharia [131] and Warwate et al. [132]. In addition to polyphenols, glucosinolates (a sulphur-containing compound) also increases under salinity stress. Yuan et al. [133] reported that total glucosinolate content increased under 100 mM NaCl treatments in radish sprouts due to the activation of genes involved in glucosinolate production. We observed that total glucosinolate content increased with root-colonizing bacterial strains singly (*Pseudomonas* and *Azotobacter*) and maximally in combination (*Pseudomonas* + *Azotobacter*). Increased glucosinolate content on inoculation with *P. fluorescens* strain (SS102) occurs under biotic stress through an increase in sulphur assimilation [134]. Supplementation with PGPR in Arabidopsis activated glucosinolate accumulation in herbivore damage as well as in insect feeding [134,135]. Contrarily, we observed a decrease in glucosinolate level with PGPR supplementation compared to salt-stressed plants, although it was higher than the control. The possible reason for increased glucosinolates under stress may be that plants’ demand for increased sulphur and glucosinolate fulfils this requirement [134]. On PGPR application, the stress is alleviated and results in a decrease in glucosinolate level. Various reports are available on the impact of PGPR on glucosinolate content or glucosinolate and abiotic stress. However, it is the first time when we are reporting glucosinolate with combined PGPR under salt stress. Glucosinolate production enhances under abiotic and biotic stress in Brassicaceae and helps in mitigating the negative effect of stress [103,136]. A study reported high glucosinolate content at low nitrogen supply and reduced glucosinolate levels at increased nitrogen supply [137]. Plants inoculated with PGPR showed lower amounts of glucosinolates when young and had higher nitrogen requirements, while in the older plants, which have a lower nitrogen requirement, increased glucosinolate levels were observed. Thus, the stress plants had a maximum requirement of glucosinolates; however, with the decrease in stress, glucosinolates content also decreased. In addition, as shown in Figure 12, the PGPR application increases S-assimilation and, thus, the formation of cysteine. Cysteine acts as precursor of both GSH and methionine. Methionine via S-adenosyl methionine (SAM) leads to excess ethylene but PGPR through ACC deaminase activity reduces ACC formation and, consequently, ethylene. Possibly, SAM could be diverted to polyamines’ formation, which can, again, lead to salt tolerance. In our study, GSH content increases maximally with PGPR application, suggesting the diversion of cysteine more towards GSH than to glucosinolate. However, we are predicting this based on the linkage in the pathway between S-assimilation, ethylene and GSH; a thorough molecular study needs to be carried out to further understand the process associated with the interaction between glucosinolates, ethylene and glutathione.

## 5. Conclusions

**Figure 13:**
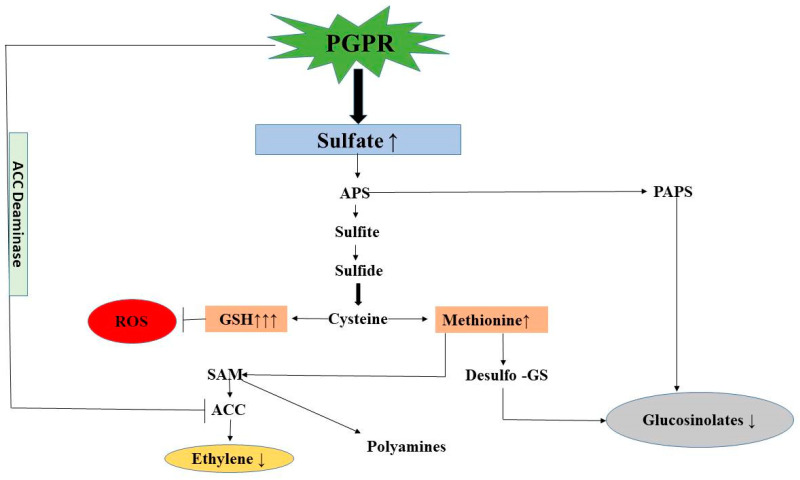
It represents a possible way of salt tolerance through PGPR application under salt stress and the explanation for the decrease in glucosinolate with PGPR compared to stress. The ↑ (upward arrow) indicates increase and ↓ (downward arrow) indicates decrease; indicates inhibition.

**Figure 14:**
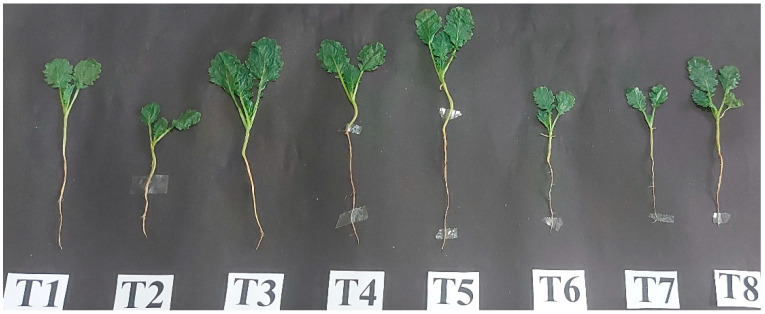
Plant photo at 30 DAS exposed to control or salt stress with or without *Pseudomonas fluorescens* or *Azotobacter chroococcum* either singly or in combination.

The severity of salt stress in plants can be reduced through the application of PGPR, which is a more eco-friendly and sustainable approach for better plant growth and yield. These PGPR are salt-tolerant and have properties that help in promoting growth in plants. Their synergistic nature under salt stress with growth-promoting properties helped the salt-stressed plants to increase photosynthesis and growth via enhancement in the antioxidant metabolism, secondary metabolites and osmolytes’ content. We have shown the role of PGPR in salt stress alleviation and growth through a model that predicts the possible interaction under stress. The reduction in stress ethylene and enhanced GSH synthesis helps in stress alleviation, which possibly explains the reduced glucosinolate content under salt stress. Further molecular studies, however, need to be conducted to unravel the glucosinolates biosynthesis pathway and more reasons for its decreased content under dual PGPR application in order to obtain a complete understanding of the scenario under salt stress. However, undoubtedly, PGPR is involved in regulating antioxidant metabolism, proline, glycine betaine, TFC and TPC for enhancing photosynthesis through ROS scavenging and ultimately increasing growth, thus providing a more sustainable approach under stress (Figure 13) The impact of PGPR is visible in Figure 14 showing plants photo at 30 DAS.

## Figures and Tables

**Table 1:** Properties of *Pseudomonas fluorescens* and *Azotobacter chroococcum*.

Bacterial Strains	Gram Reaction	Cell Shape	IAA (µg mL^−1^)	ACC Deaminase Production (µM α Ketobutyrate mg^−1^ Protein h^−1^)	Siderophore Production CAS Agar Plate Assay Zone Formation and Zone Production in (mm)	Ammonia Production	HCN Production	Phosphate Solubilisation
*Pseudomonas fluorescens*	Gram-negative	Short rod shaped	86.8 ± 1.79	210 ± 0.42	+ve 21.4 ± 1.3	+	+	+
*Azotobacter chroococcum*	Gram-negative	Oval shaped	79.4 ± 2.17	193 ± 1.65	+ve 13.2 ± 1.7	+	+	+

**Table 2:** Effect on root length, shoot length, height of plant, root fresh and dry biomass, shoot fresh and dry biomass, number of leaves and leaf area in mustard cultivar Pusa Jagannath exposed to control or salt stress with or without *Pseudomonas fluorescens* (PGPR1) or *Azotobacter chroococcum* (PGPR2) either singly or in combination. Data are presented as treatments mean ± SE (*n* = 4). Data followed by same letter are not significantly different by LSD test at *p* < 0.05.

Treatment	Root Length (cm)	Shoot Length (cm)	Height of Plant (cm)	Root Biomass (g Plant^−1^)		Shoot Biomass (g Plant^−1^)		No. of Leaves	Leaf Area (cm^2^)
				Fresh	Dry	Fresh	Dry		
Control	6.667 ± 0.37 ^d^	10.23 ± 0.77 ^d^	16.900 ± 0.84 ^d^	0.08 ± 0.00 4 ^d^	0.017 ± 0.001 ^c^	0.849 ± 0.065 ^d^	0.110 ± 0.012 ^d^	8.333 ± 4.49 ^d^	7.10 ± 0.63 ^d^
100 mM NaCl	3.530 ± 0.281 ^g^	6.633 ± 0.58 ^g^	10.163 ± 0.62 ^g^	0.04 ± 0.002 ^g^	0.009 ± 0.0009 ^g^	0.38 ± 0.0335 ^g^	0.047 ± 0.0056 ^g^	3.667 ± 1.700 ^g^	4.63 ± 0.105 ^g^
PGPR1	7.683 ± 0.56 ^b^	13.40 ± 0.94 ^b^	21.063 ± 0.99 ^b^	0.10 ± 0.006 ^b^	0.027 ± 0.002 ^b^	1.287 ± 0.046 ^b^	0.203 ± 0.019 ^b^	12.667 ± 3.300 ^b^	10.6 ± 0.304 ^b^
PGPR2	7.013 ± 0.486 ^c^	12.1 ± 0.85 ^c^	20.003 ± 0.91 ^c^	0.09 ± 0.005 ^c^	0.012 ± 0.001 ^c^	1.03 ± 0.071 ^c^	0.172 ± 0.0176 ^c^	10.000 ± 2.449 ^c^	9.19 ± 0.961 ^c^
PGPR (1 + 2)	8.560 ± 0.662 ^a^	15.90 ± 0.99 ^a^	24.460 ± 1.055 ^a^	0.19 ± 0.008 ^a^	0.038 ± 0.003 ^a^	1.56 ± 0.072 ^a^	0.357 ± 0.0246 ^a^	17.333 ± 2.494 ^a^	14.03 ± 0.74 ^a^
100 mM NaCl + PGPR1	5.220 ± 0.362 ^e^	8.067 ± 0.66 ^e^	13.287 ± 0.71 ^e^	0.07 ± 0.004 ^e^	0.013 ± 0.001 ^c^	0.654 ± 0.07 ^e^	0.077 ± 0.0064 ^e^	6.000 ± 2.160 ^e^	5.810 ± 0.76 ^e^
100 mM NaCl + PGPR2	4.633 ± 0.415 ^f^	7.100 ± 0.58 ^f^	11.933 ± 0.67 ^f^	0.06 ± 0.003 ^f^	0.010 ± 0.001 ^d^	0.51 ± 0.057 ^f^	0.064 ± 0.0074 ^f^	4.667 ± 3.859 ^f^	4.83 ± 0.348 ^f^
100 mM NaCl + PGPR (1 + 2)	6.300 ± 0.505 ^d^	9.167 ± 0.72 ^d^	16.07 ± 0.92 ^d^	0.08 ± 0.04 ^d^	0.016 ± 0.002 ^c^	0.807 ± 0.041 ^d^	0.102 ± 0.09 ^d^	7.667 ± 4.497 ^d^	6.797 ± 0.9 ^d^

**Table 3:** Effect on total glucosinolate content, total phenolic content, total flavonoid content and carotenoids content in mustard cultivar Pusa Jagannath exposed to control or salt stress with or without *Pseudomonas fluorescens* (PGPR1) or *Azotobacter chroococcum* (PGPR2) either singly or in combination. Data are presented as treatments mean ± SE (*n* = 4). Data followed by same letter are not significantly different by LSD test at *p* < 0.05.

Treatments	Total Glucosinolate Content Total Glucosinolate (μmol g^−1^ FW)	Total Phenolic Content (mg GAE g^−1^ FW)	Total Flavonoid Content (mg Rutin g^−1^ FW)	Carotenoids (mg g^−1^ FW)
Control	19.189 ± 1.18 ^g^	07.211 ± 0.33 ^f^	08.799 ± 0.43 ^f^	0.885 ± 0.045 ^d^
Salt	31.204 ± 2.12 ^a^	10.415 ± 0.76 ^e^	12.056 ± 0.82 ^e^	0.408 ± 0.011 ^g^
PGPR1	24.538 ± 1.44 ^e^	13.158 ± 0.84 ^c^	15.694 ± 0.91 ^b^	1.052 ± 0.090 ^b^
PGPR2	23.746 ± 1.26 ^f^	12.189 ± 0.73 ^d^	13.444 ± 0.77 ^d^	0.949 ± 0.082 ^c^
PGPR(1 + 2)	27.906 ± 1.69 ^c^	15.416 ± 1.09 ^a^	18.653 ± 1.16 ^a^	1.217 ± 0.181 ^a^
Salt + PGPR1	25.608 ± 1.53 ^d^	12.025 ± 0.73 ^d^	14.883 ± 0.79 ^c^	0.610 ± 0.061 ^e^
Salt + PGPR2	24.142 ± 1.31 ^e^	10.480 ± 0.81 ^e^	13.606 ± 0.71 ^d^	0.526 ± 0.033 ^f^
Salt + PGPR(1 + 2)	28.817 ± 1.78 ^b^	14.204 ± 0.94 ^b^	15.347 ± 1.01 ^b^	0.838 ± 0.043 ^d^

## Data Availability

All figures and tables are inserted in the text.

## Supplementary Materials

The following supporting information can be downloaded at: https://www.mdpi.com/article/10.3390/plants12040705/s1.
