# Supplementary material for: Palliating Salt Stress in Mustard through Plant-Growth-Promoting Rhizobacteria: Regulation of Secondary Metabolites, Osmolytes, Antioxidative Enzymes and Stress Ethylene
Source: Plants (Basel). 2023 Feb 5;12(4):705. doi: 10.3390/plants12040705 (PMC9963382; doi:10.3390/plants12040705)
Supplement: Supplementary file 1 [file plants-12-00705-s001.zip › plants-2099681-supplementary.pdf]

Palliating salt stress in mustard through plant growth promoting rhizobacteria: regulation of secondary metabolites, osmolytes, antioxidative enzymes and stress ethylene

Varisha Khan, Shahid Umar * and Noushina Iqbal *

Department of Botany, Jamia Hamdard, New Delhi-110062,India
* Correspondence: sumer@jamiahamdard.ac.in (S.U.); naushina.iqbal@gmail.com or noushinaiqbal@jamiahamdard.ac.in (N.I.)

Materials and Methods

Plant material and growth conditions

Mustard (*Brassica juncea* L. var. Pusa Jagannath VSL-5) seeds obtained from Indian Institute of Agriculture Sciences (IARI) , New delhi. Healthy seeds similar in shape and size were surface sterilized with 0.01% $HgCl_2$ followed by four times rinsing with double distilled water. Washed seeds were sown into earthen pots(containing 8 kg of soil during the spring season of 2021 at the herbal garden of Jamia Hamdard, New Delhi, India (extending from 28.51344 N and 77.2475 E at an elevation of 782 feet above sea level). According to Köppen's classification, the climate was humid continental (Dfb), with an average temperature of 25 °C and mean annual precipitation of 990.9 mm, mostly falling from June to September. The experiment's design used a randomised block layout. Using the recommended basal dosages of N, P, and K for 120 kg/ha, 80 kg/ha, and 60 kg/ha respectively, the soil used in pots was uniformly blended with NPK at the time of pre sowing and 15 days after the emergence of leaves. Urea, potash, and single superphosphate (SSP) were the sources of N,P and K.

Procurement of Bacterial strains

Plant growth promoting rhizobacteria (PGPR) bacterial strains *Pseudomonas flourescens* (NAIMCC-B-00340) and *Azotobacter chroococcum* Beijerinck 1901 (Accession No.MCC 2351) were obtained from National Bureau of Agriculturally Important Microorganisms (NBAIM) Mau, and National Centre for Cell Science(NCCS), Pune respectively. Selection of these strains were based on their growth promoting properties. Nutrient broath was used for making an overnight culture of these strains.

Inoculation of PGPR, NaCl treatment and experimental design

Healthy and sterilized seeds were dipped in two strains *Pseudomonas flourescens*, *Azotobacter chroococcum* and combination of both strains for two hours using 1% guar gum powder as adhesive to deliver roughly 10^8 cells per seed. Also, the uninoculated seeds were just immersed in sterile water serving as control. The inoculated and uninoculated seeds (25seeds/pot) were sown in earthen pots containing. After the emergence of leaves,salt stress (100 mM) was given with 600mL per pot. Thereby, the experiment was comprised of 8 treatments with three replicates each

T1 (Control, with Nutrient broath but without salt and PGPR),

T2 (100 mM NaCl),

T3 (*P. flourescens*),

T4 (*A. chroococcum*),

T5 (*P. flourescens+A. chroococcum*),

T6 (100 mM NaCl t+ *P. flourescens*),

T7 (100 mM NaCl + *A.chroococcum),*

T8 (100 mM NaCl + *P. flourescens + A. chroococcum)*

Analysis of morphological parameters

Biological samples from all treatments were taken randomly at 30 Days after sowing (DAS) to measure root-shoot length, plant height, fresh and dry weight of root and shoot, number of leaves, leaf area. The plants were uprooted gentally and root, shoot length measured with metric scale and vernier caliper. Fresh weights of root and shoot were recorded utilizing an electric analytical balance (Vibra DJ 1505/Denver Instrument, APX 200). The samples were then kept in an oven (Scientific Systems 1.01) at 65 °C for 72 hours to dry out before their constant weight and dry weights were calculated using an electronic balance (Denver Instrument, APX 200).

Biochemical Parameters

Measurement of photosynthetic parameters and soluble protein

Chlorophyll a,b,total chlorophyll measured in fresh leaves by adopting Hiscox and Israelstam (1979) method. Fresh leaves weighing 100 mg were cut and placed in a test tube containing 7mL of dimethy sulfoxide (DMSO). For percolating the pigments, the test tubes were covered with aluminium foil and incubated at 65 °C for 40 minutes. The final volume was reached to 10 mL by adding DMSO. The chlorophyll content was then measured by taking 3 mL of plant extract and recorded absorbance at 645, 663 using UV–Vis Spectrophotometer (Model 119,Systronics, India). Blank was measured by taking only 3mL DMSO without sample. The chlorophyll content was expressed as mg g^{-1} fresh weight (mg g^{-1} FW).

The Total soluble protein was measured in fresh leaves by Bradford (1976) method. Fresh leaves weighed 0.1g was homogenised in 2 mL of 0.1 M phosphate bufer pH 6.8 in pre chilled morter pestle and kept in ice during homegenisation, centrifuged at 5000×g for 10 min at 4 °C. To the supernatant, equal amount of pre-chillled 20% TCA was added and then centrifuged again at 3300×g. Supernatant was discarded and pellet was washed with acetone and then dissolved in 1 mL of 0.1 N NaOH. To 1ml aliquot, 5ml of Bradford reagent was added, tubes covered with aluminium foil and kept in dark for optimum colour development. By using the bovine serum albumin (BSA) (Sigma-Aldrich) as a standard and taking the absorbance at 595 nm, the amount of soluble protein was calculated and expressed as mg g^{-1} FW.

Plants net photosynthesis, stomatal conductance and intercellular CO_2 were measured with infrared gas analyser (IRGA, LICOR-6400XT) in proper expanded leaves under proper bright sunlight. During the measurement photosynthetically active radiation (PAR) was µmol $m^{-2}s^{-1}$ and atmospheric CO^2 concentrations was 390 ± 5 µmol mol^{-1} (i.e., at light saturating intensity).

Estimation of proline, total soluble sugar and glycinebetaine

Proline was estimated by applying Bates et al. (1973) method. 0.3g of fresh leaves were weighed and homogenised in 10 ml of 3% of sulphosalicylic acid and centrifuged at 8000×g for 10 min. To the 0.2mL of supernatant, 2 mL of acid ninhydrin and 2 mL of glacial acetic acid was added and was kept at 100 °C for 1h in water-bath. Transferring tubes immediately to the cold bath allowed the product to develop, which was then extracted by adding 4 mL of toluene and vortexing. L-proline (Sigma Aldrich) was used as a standard and absorbance was taken at 520 nm by taking upper layer of toluene and expressed as mg g^{-1} FW.

Total soluble sugar (TSS) was estimated by method of Dey (1990). Fresh leaves weighing 0.1g were chopped and put in test tube containing 10mL of 70% ethanol. Test tubes were then incubated at 60 °C in oven for 1 hour twice. Final volume was reached upto 25mL by adding double distilled water. To 1mL of supernatant, 1mL of 5% phenol and 5mL conc. Sulphuric acid was added and product was cooled in order for exothermic reaction to occur. D-Glucose (Sigma-Aldrich) was used as a standard and absorbance was taken at 485nm and expressed as mg g^{-1} FW.

Glycine betaine (GB) content was estimated by Grieve and Grattan (1983) method. Fresh leaves weighing 0.5g were homogenized in 5 mL of 0.5% toluene followed by filteration and supernatant was collected and stored at 4^0C. After 12h, 1mL of this filterate was mixed with 1mL of sulphuric acid. To every 0.5 mL mixture, 200 µL of potassium tri-iodide was added. Immediately cool down on ice and add 2.8mL of chilled double deionized water and 5 mL of 1, 2-di-chloroethane. Organic layer (lower layer) was utilized for recording absorbance 365 nm.

Estimation of Phenol, flavonoid, carotenoids and glucosinolate content

Total phenolic content (TPC) was measured by Ainsworth and Gillespie (2007) by using Folin-Ciocalteu (F-C) reagent. 0.25g fresh leaves weighed and homogenized in 2mL pre chilled methanol(95%) followed by centrifugation at 12500 g for 10 minutes and supernatant was collected in fresh eppendorf tube. To every sample, 100ul of this supernatant and 200ul of 10% FC reagent was added and vortexed thoroughly. In addition to this, 800ul of 700mM sodium carbonate was added to every tube and then tubes were kept for incubation for 90 minutes at room temperature. A standard curve was prepared by taking different concentrations of gallic acid. Prepared extract was used for absorbance at 765nm and phenolic content was expressed as mgGAE g^{-1} FW.

Total Flavonoid content (TFC) was estimated by Aluminium trichloride complex forming assay. Test sample 100ug/mL was prepared in pre chilled methanol and centrifuged at 12500 g for 10 minutes. 1mL of test sample was diluted with 4 mL of distilled water followed by addition of 0.3mL of 5% $NaNO_2$. 0.3 mL of 10% $AlCl_3$ was added after six minutes, and the mixture was let to stand for six more minutes.To this mixture, 2Ml NaOH (1mM) was added

and finally reaction mixture was reached to 5mL by diluting with distilled water and left for 15 minutes at room temperature. A standard curve was prepared by using different concentration of rutin(10-100ug/mL), absorbance was taken at 510nm and expressed as mg rutin g^{-1} FW.

The Hiscox and Israelstam (1979) method was used to quantify the carotenoid content in fresh leaves. It measured absorbance at 480 and 510 nm and presented the results as mg g^{-1} FW.

Total glucosinolate content was estimated by previously published protocols with slight modifications (kiddle et al,2001;Doheny-Adams et al., 2017). 1 gram fresh leaf samples weighed and macerated in 5mL of (70:30) Methanol:water. To stop the activity of myrosinase, the extract was then heated on a water bath for 25 minutes at 70ºC. The extract was transferred into falcon tubes followed by centrifugation at 8000rpm for 15 minutes. Collected supernatant containing total glucosinolates was then evaporated to dryness at 50^0C in a rotavapour. To 100ul of methanolic extract , 0.3mL of double distilled water and 3mL of 2mM sodium tetrachloropalladate was added. The mixture was left for incubation for 60 minutes at 25 ± 5^0C. Extract was taken for absorbance at 425nm and expressed as µmol

g^{-1}FW.

Estimation of Antioxidant enzymes

Enzyme Extraction

Fresh leaves weighing 0.5g homogenized in 50mM sodium phosphate buffer(pH 7.0) containing 1mM ethylenediaminetetraacetic acid (EDTA) and 2% PVPP (polyvinylpolypyrrolidone) using pre chilled morter and pestle. This homogenate was centrifuged at 13,000×g at 4 °C for 20 minutes and supernatant collected was carried out for antioxidant enzyme assays; SOD, CAT, APX and GR.

Superoxide Dismutase

Superoxide dismutase(SOD) activity was measured by Beuchamp and Fridovich, 1971. To 100ul enzyme extract , 3mL reaction mixture containing (100mM phosphate buffer (pH 7.8), 0.1mM EDTA, 13mM methionine, 2.25mM NBT, 60uM riboflavin) was added. The tubes were placed under 40W light for 15 minutes. Enzyme activity was measured at 560nm and expressed as U mg^{-1} Protein min^{-1}

Catalase

Catalase (CAT) activity was estimated by Beers and Sizeir,1952. 3mL catalse reaction mixture containing (100mM phosphate buffer(pH 7.0), 0.1mM EDTA, 20mM H_2O_2) was mixed with 50ul of enzyme extract. Monitor the decrease in the absorbance at 240 nm and quantify by its extinction coefficient of 0.036 $mM^{-1}cm^{-1}$ and expressed as U mg^{-1} Protein min^{-1}

Ascorbate Peroxidase

Ascorbate peroxidase (APX) activity was determined by Nakano and Asada (1981). 3 mL of assay mixture consisting (50Mm phosphate buffer(pH 7.0), 500mM ascorbic acid, 1mM H_2O_2) was mixed with 100µl enzyme extract. APX activity was measured as decrease in absorbance at 290nm and expressed as U mg^{-1} Protein min^{-1}

Glutathione Reductase

Glutathione Reductase (GR) activity was assayed by Jablonski and Anderson, 1978 method. To 3mL of reaction mixture comprising (100mM phosphate buffer (pH 7.5), 1mM oxidized glutathione, 1mM EDTA, 0.1mM NADPH) , 50ul enzyme extract was added. Oxidation of NADPH was followed by monitoring the decrease in absorbance per min at 340 nm. Enzyme activity was expressed as U mg^{-1} Protein min^{-1}

Content of reduced glutathione

The content of GSH in leaves was determined by the methods of Anderson (1985). 500 g of fresh leaves were homogenized in 5% sulphosalicylic acid (2.0 mL) under cold conditions. The homogenate obtained was centrifuged for 10 min. at 10, 000 × g. Supernatant (0.5 mL), phosphate buffer (0.6 mL,100 mM, pH 7.0) and 5, 5, dithiobis-2-nitrobenzoic acid (DTNB, 40 µl) were added. The absorbance was recorded at 412 nm after 2 min of incubation.

Determination of Lipid peroxidation , H_2O_2 content and ethylene evolution

MDA content (measure of lipid peroxidation) was determined by method provided by Zhou and Leul (1998). Fresh leaves weighed 0.5g was homogenized in 5mL of 1%TCA followed by centrifugation at 8000rpm for 10 minutes. To every 0.5mL of supernatant, 2mL of 20%TCA containing 0.5% TBA was added. Resultant mixture was heated for 30 minutes at 95^0C and then immediately cooled on Ice bath. Product was again centrifuged at 8000rpm for 15 minutes , supernatant collected and was used for taking absorbance at 532nm, 600nm and 450nm.

H_2O_2 hydrogen peroxide content was measured by Velikova et al. (2000). 0.5g of fresh leaves were homogenized in 5ml of 0.1% TCA in pre chilled morter pestle followed by centrifugation at 12,000 rpm for 15 min. To 0.5mL of supernatant, 0.5mL of 10 mM potassium phosphate buffer (pH 7.0) and 1 mL of 1 M potassium iodide (KI) solution was added. H_2O_2 content was measured at 390nm.

Ethylene evolution was measured using a gas chromatograph (Sehar et al. (2021). Level of ethylene was estimated using gas chromatograph by cutting 500 mg of plant leaf into small pieces and placed into 30 mL tubes containing moist paper for minimizing the evaporation from the tissues and stoppered with secure rubber caps and kept in light for 2 h under the same condition used for the plant growth. An earlier experiment showed that 2 h of incubation time was appropriate for ethylene detection without the interference of wound induced ethylene, which started after 2 h of leaf incubation. A 1 mL of gas samples from the tubes were taken by a hypodermic syringe and assayed on a gas chromatograph (Nucon 5700, New Delhi, India) endowed with a 1.8 m Porapack N (80-100 mesh) column, a flame ionization detector and data station. Nitrogen was used as the carrier gas. The flow rates of hydrogen, nitrogen and oxygen were 30, 30 and 300 mL min^{-1}, respectively; the detector was set at 150° C. Ethylene was detected based on retention time and measured by comparison with peaks from standard ethylene concentration.

Cellular damage detection and viability measurement in mustard roots by CLSM

Confocal laser scanning microscopy for Salt and PGPR treated roots was performed to check membrane damage and cell viability. Fresh roots were cut into small pieces and followed dual staining with 30 µM propidium iodide (PI; Hi Media, India) and 10 µM acridine orange (AO; Hi Media, India) solution for 15 min. After being treated with a dye mixture, mustard roots were then rinsed with phosphate buffer (0.1 M, pH 7.0), and finally mounted on glass slides. Stained Roots were inspected for both live and dead tissues under confocal laser scanning microscope Leica Microsystems TCS-SP5. Dead and live tissues showed PI and AO staining respectively. Control roots were used for assessing the difference between treated and untreated roots.

Determination of visible leaf damage by superoxide through histochemical staining

Histochemical staining of superoxide ion ($O2^{\cdot -}$) was performed in the leaves of mustard plant by the method of kumar et al. (2014) with few modifications. To stain leaves nitro blue tetrazolium (NBT) was used. Freshly prepared NBT solution was used for detecting ($O2^{\cdot -}$). 0.2g of NBT was dissolved in 100mL of sodium phosphate buffer to prepare the NBT solution (50Mm, pH 7.5). Leaf samples were then immersed in NBT solution and incubated for an overnight period at room temperature. The samples were then boiled in absolute ethanol for 20 min and then photographs were taken.

Compatibility assay

Compatibility assay was performed by well diffusion method. To check their compatibility under in vitro conditions 50 µl culture of *P.flourescens* was spreaded on Nutrient agar (NA) plate and 50 µl culture of *A.chroococcum* was filled in the well formed on plate and later NA plate kept at 28 ± 2 °C for 24 hours in incubator. Presence or absence of inhibition zone around the well would confirm the antagonism and synergism respectively between two PGPR.

Statistical analysis

Data collected from the completely randomized block design experiments were analyzed statistically using analysis of variance (ANOVA) by SPSS 17.0 for windows and presented as mean ± SE (n = 4). The least significant difference was calculated for the significant data at $p< 0.05$. The PCA and Pearson correlation were carried out using Origin Pro software. To create biplots, the first two components (PC1 and PC2) showing the maximum variance in the datasets were considered.

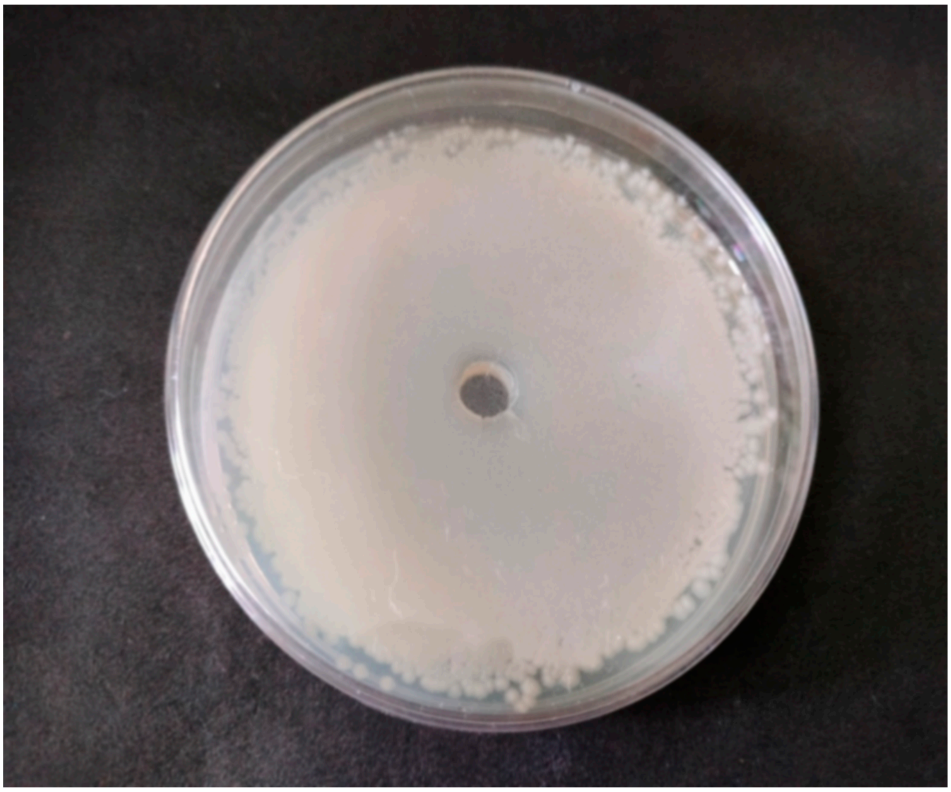

Synergism between *P. flourescens* (spreaded) and *A. chroococcum* (inside well).
